# Supplementary material for: Solvent tuning of photochemistry upon excited-state symmetry breaking
Source: Nat Commun. 2020 Apr 21;11:1925. doi: 10.1038/s41467-020-15681-3 (PMC7174366; doi:10.1038/s41467-020-15681-3)
Supplement: Supplementary file 1 — Supplementary Information [file 41467_2020_15681_MOESM1_ESM.pdf]

SUPPLEMENTARY INFORMATION

Solvent Tuning of Photochemistry upon Excited-State Symmetry Breaking

Dereka *et al.*

SUPPLEMENTARY METHODS

Synthesis

Synthesis of D, Q and O with methyl substituents

Chemicals were purchased from Acros Organics, TCI America and Sigma-Aldrich, and used without further purification. NMR spectra were recorded at 500 MHz for ^{1}H and 125 MHz for ^{13}C on a Bruker Advance III HD spectrometer. Data for ^{1}H NMR are reported as follows: chemical shift in units of parts per million (ppm) from tetramethylsilane (TMS) using the residual non-deuterated signal of $CDCl_3$ (δ_H = 7.26 ppm) as internal reference; multiplicities are reported using the following abbreviations: s - singlet, d - doublet. Data for ^{13}C NMR are reported in ppm from TMS using the central peak of the solvent as reference ($CDCl_3$, δ_C = 77.16 ppm). Mass-spectroscopy data were acquired on 4800 MALDI TOF (Sciex, Inc.) in SPA/60% acetonitrile/0.1% TFA in reflector positive mode with Nd:YAG laser (355 nm @ 200 Hz).

D synthesis was described in our previous work [1].

Q was obtained in two consecutive steps according to the procedure described in [2].

Chloro-[2-[4-(dimethylamino)phenyl]ethynyl]zinc. On the first step, 4-ethynyl-*N,N*-dimethylaniline (2.18 g, 15 mmol) was dissolved in 30 mL of dry THF in a three-neck round-bottom flask under argon atmosphere and cooled down to -78°C on an acetone-dry ice bath. Subsequently, *n*-BuLi (6.63 mL, 1 eq) was added to the reaction mixture, followed by the suspension of anhydrous $ZnCl_2$ (2.46 g, 1.2 eq) in 20 mL of dry THF. The mixture was allowed to react for 1 hour under constant stirring. The obtained product was directly used for the next step.

2,4-bis(4'-dimethylaminophenyl)ethynyl-6-chloro-1,3,5-triazine (Q). To the reaction mixture obtained on the previous step (3 eq assuming 100 % yield on step 1) and stirred at room temperature and under argon atmosphere, 2,4,6-trichloro-1,3,5-triazine (0.92 g, 1 eq) was added. Tetrakis(triphenylphosphine)palladium(0) (0.29 g, 0.05 eq) was subsequently added to the reaction mixture, and the reaction was allowed to proceed for 3 hours at room temperature and constant stirring. After that, the reaction was quenched by adding a piece of ice. The organic phase was removed under vacuum, and the mixture was extracted with chloroform (5x20 mL). The extract was dried over the anhydrous $MgSO_4$ overnight, and concentrated under vacuum. The crude product was subsequently purified using column chromatography (silica gel, chloroform). The main impurity is the mono-substituted triazine. The target compound was obtained as red powder (0.636 g, 32%). ^{1}H NMR (500 MHz, $CDCl_3$): 3.06 (s, 12H), 6.68 (d, 4H), 7.68 (d, 4H). ^{13}C NMR (125 MHz, $CDCl_3$): 40.03, 87.03, 99.67, 111.37, 111.56, 135.34, 151.88, 161.27, 172.24. MALDI-TOF MS m/z = 400.09 (M-1), 401.08 (M), 402.09 (M+1).

O was obtained in two consecutive steps according to the procedure described in [2].

Chloro-[2-[4-(dimethylamino)phenyl]ethynyl]zinc. On the first step, 4-ethynyl-*N,N*-dimethylaniline (0.671 g, 4.62 mmol) was dissolved in 25 mL of dry THF in a three-neck round-bottom flask under argon atmosphere and cooled down to -78°C on an acetone-dry ice bath. Subsequently, *n*-BuLi (2.04 mL, 1 eq) was added to the reaction mixture, followed by the suspension of anhydrous $ZnCl_2$ (0.758 g, 1.2 eq) in 15 mL of dry THF. The mixture was

allowed to react for 1 hour under constant stirring. The obtained product was directly used for the next step.

2,4,6-tris(4'-dimethylaminophenyl)ethynyl-1,3,5-triazine (O). To the reaction mixture obtained on the previous step (3.5 eq assuming 100 % yield on step 1) and stirred at room temperature and under argon atmosphere, 2,4-bis(4'-dimethylaminophenyl)ethynyl-6-chloro-1,3,5-triazine (**Q**, 0.53 g, 1 eq) was added. Tetrakis(triphenylphosphine)-palladium(0) (0.08 g, 0.05 eq) was subsequently added to the reaction mixture. The reaction was brought to reflux and allowed to proceed for 5 hours with constant stirring. After that, the reaction was quenched by adding a piece of ice. The organic phase was removed under vacuum, and the mixture was extracted with chloroform (5x20 mL). The extract was dried over the anhydrous $MgSO_4$ overnight, and concentrated under vacuum. The crude product was subsequently purified using column chromatography (silica gel, hexanes-ethyl acetate 1:1 v/v). The main impurity is the disubstituted precursor. The target compound was obtained as yellow powder (0.233 g, 35%). ^1H NMR (500 MHz, $CDCl_3$): 3.06 (s, 18H), 6.67 (d, 6H), 7.64 (d, 6H). ^{13}C NMR (125 MHz, $CDCl_3$): 40.03, 87.12, 101.44, 111.37, 113.73, 135.51, 151.79, 161.05, 171.15. MALDI-TOF MS m/z = 510.19 (M-1), 511.21 (M), 512.22 (M+1).

Synthesis of D and O with butyl substituents.

Chemicals used in the current work were purchased from either Sigma-Aldrich, TCI Europe or ABCR and used without further purification. NMR spectra were recorded at 600 MHz for ^1H and 150 MHz for ^{13}C on a Bruker Avance III HD spectrometer and at 400 MHz for ^1H and 100 MHz for ^{13}C on a Bruker Avance DRX-400 spectrometer. Data for ^1H NMR are reported as follows: chemical shift in units of parts per million (ppm) from tetramethylsilane (TMS) using the residual non-deuterated solvent signal of CD_2Cl_2 (δ_H = 5.32 ppm) or $CDCl_3$ (δ_H = 7.26 ppm) as internal reference. Multiplicities are reported by using the following abbreviations; s: singlet; d: doublet; t: triplet; m: multiplet; J: coupling constants in Hertz (Hz). ^{13}C NMR data are reported in ppm from TMS using the central peak of the solvent as reference (CD_2Cl_2: δ_C = 53.84 ppm, CDCl3: δ_C = 77.16 ppm); the multiplicity with respect to H (s = quaternary C, d = CH, t = CH_2, q = CH_3) is deduced from attached proton test (APT) experiments.

HRESIMS spectra (m/z 50-1900) were obtained on a maXis ESI-Qq-TOF mass spectrometer (Bruker Daltonics, Bremen, Germany) in the positive-ion mode by direct infusion. The sum formulas of the detected ions were determined using Bruker Compass DataAnalysis 4.0 based on the mass accuracy (m/z ≤ 5 ppm) and isotopic pattern matching (SmartFormula algorithm).

The alkyne-precursor *N,N*-dibutyl-4-ethynyl-benzenamine was synthesized according to ref. [3,4].

4-[4-(*N,N*-Dibutylamino)phenyl]-2-methyl-3-butyn-2-ol. *N,N*-Dibutyl-4-iodo-aniline (4.636 g, 14.0 mmol, 1.0 eq) was dissolved in dry trimethylamine (135 mL) and degassed using argon. 2-Methyl-3-butyne-2-ol (1.648 g, 19.6 mmol, 1.4 eq), bis(triphenylphosphine)palladium(II) dichloride (0.197 g, 0.3 mmol, 2 mol%) and copper(I) iodide (0.053 g, 0.3 mmol, 2 mol%) were added sequentially under a counter flow of argon. The reaction mixture was stirred at 40°C overnight. The progress of the reaction was monitored by TLC. After full conversion, the reaction mixture was filtered over a pad of silica to remove catalyst remains. The solid residue was washed with DCM (300 mL). The

combined organic layers were washed with water (3x100 mL) and brine (100 mL), dried over sodium sulfate and the volatile compounds were removed under reduced pressure. The product was purified by column chromatography (silica, light petrol:ethyl acetate = 0-10%) yielding 3.292 g (82%) of an orange liquid.
^{1}H-NMR (400 MHz, $CDCl_3$): δ = 7.24 (d, J = 9.0 Hz, 2H, Ar-H), 6.52 (d, J = 9.0 Hz, 2H, Ar-H), 3.25 (t, J = 7.7 Hz, 4H, -N-CH_2-), 2.02 (s, 1H, -OH), 1.60 (s, 6H, -C-CH_3), 1.55 (m, 4H, -N-CH_2-CH_2-), 1.34 (m, 4H, -CH_2-CH_3), 0.95 (t, J = 7.3 Hz, 6H, -CH_2-CH_3) ppm.

***N,N*-Dibutyl-4-ethynylbenzenamine.** 4-[4-(*N,N*-Dibutylamino)phenyl]-2-methyl-3-butyn-2-ol (3.29 g, 11.5 mmol, 1.0 eq) was dissolved in dry toluene (150 mL) and freshly powdered KOH (0.90 g, 16.0 mmol, 1.4 mmol) was added. The reaction mixture was degassed with argon and heated to reflux. The progress of the reaction was monitored by GC-MS. After full conversion the yellow reaction mixture was filtered through a pad of silica. The solid remains were washed with toluene. The solvents were removed in vacuum yielding 2.52 g (96%) of a dark yellow liquid.
^{1}H-NMR (400 MHz, $CDCl_3$): δ = 7.21 (d, J = 9.0 Hz, 2H, Ar-H), 6.53 (d, J = 9.0 Hz, 2H, Ar-H), 3.27 (t, J = 7.5 Hz, 4H, -N-CH_2-), 2.96 (s, 1H, yne-H), 1.56 (m, 4H, -N-CH_2-CH_2-), 1.34 (m, 4H, -CH_2-CH_3), 0.96 (t, J = 7.5 Hz, 6H, -CH_2-CH_3) ppm.

D was synthesized in two consecutive steps according to a previously published protocol described in [1,2].

Chloro-[2-[4-(*N,N*-dibutylamino)phenyl]ethynyl]zinc. *N,N*-Dibutyl-4-ethynylbenzenamine (275 mg, 1.20 mmol, 1.0 eq) was dissolved in 6 mL of dry THF in a Schlenck tube under argon atmosphere and cooled to -78°C in an acetone-dry ice bath. Subsequently, *n*-BuLi (0.50 mL, 1.26 mmol, 1.05 eq) was added to the reaction mixture, followed by a solution of anhydrous $ZnCl_2$ (196 mg, 1.44 mmol, 1.2 eq) in dry THF (4 mL). The solid $ZnCl_2$ was pre-dried by melting in high vacuum using a heat gun. The mixture was allowed to react for 1 hour under constant stirring. The obtained product was directly used for the next step.

4-[2-(4,6-Dichloro-1,3,5-triazine-2-yl)ethynyl]-*N,N*-dibutylaniline (D). To the reaction mixture obtained in the previous step and stirred at room temperature and in argon atmosphere, 2,4,6-trichloro-1,3,5-triazine (221 mg, 1.2 mmol, 1 eq assuming 100% yield on the step 1) was added. Tetrakis(triphenylphosphine)palladium(0) (69 mg, 0.06 mmol, 5 mol%) was subsequently added to the reaction mixture resulting in an instantaneous color change to red. The reaction was allowed to proceed for 1 hour at room temperature under constant stirring, until TLC showed full consumption of starting materials. The reaction mixture was flashed over a pad of silica using light petrol and diethyl ether as eluent. The raw product was purified by column chromatography (silica, light petrol:diethyl ether = 0-10%) giving 316 mg (70%) of a red oil.
^{1}H-NMR (600 MHz, CD_2Cl_2): δ = 7.52 (d, J = 9.1 Hz, 2H, Ar-H), 6.63 (d, J = 9.1 Hz, 2H, Ar-H), 3.34 (t, J = 7.8 Hz, 4H, -N-CH_2-), 1.59 (m, 4H, -N-CH_2-CH_2-), 1.37 (m, 4H, -CH_2-CH_3), 0.97 (t, J = 7.4 Hz, 6H, -CH_2-CH_3) ppm.
^{13}C-NMR (150 MHz, CD_2Cl_2): δ = 171.5 (s, triazine), 162.3 (s, triazine), 151.1 (s, C-N), 136.2 (d, -C-C-C≡C), 111.8 (d, C-C-N), 104.0 (s, C-C≡C-triazine), 103.6 (s, -C≡C-triazine), 87.8 (d, -C≡C- triazine), 51.1 (t, -N-CH_2-), 29.6 (t, -N-CH_2-CH_2-), 20.6 (t, -CH_2-CH_3), 14.1 (q, -CH_2-CH_3) ppm.
HRMS (ESI): $[M + H]^+$ m/z calcd. for $C_{19}H_{23}Cl_2N_4^+$ 377.1294; found 377.1291.
T_m = 81 °C

O was synthesized in two consecutive steps according to a modified procedure from literature.[5,6]

Chloro-[2-[4-(*N,N*-dibutylamino)phenyl]ethynyl]zinc. *N,N*-Dibutyl-4-ethynyl-benzenamine (413 mg, 1.80 mmol, 1.0 eq) was dissolved in 10 mL of dry THF in a Schlenck tube in argon atmosphere and cooled down to -78°C in an acetone-dry ice bath. Subsequently, *n*-BuLi (0.76 mL, 1.89 mmol, 1.05 eq) was added to the reaction mixture, followed by a solution of anhydrous $ZnCl_2$ (294 mg, 2.16 mmol, 1.2 eq) in dry THF (8 mL). The solid $ZnCl_2$ was pre-dried by melting in high vacuum using a heat gun. The mixture was allowed to react for 1 hour under constant stirring. The obtained product was directly used for the next step assuming 100% yield (1.0 eq).

Tris-4-[2-(4,6-dichloro-1,3,5-triazine-2-yl)ethynyl]-*N,N*-dibuthylaniline (O). 2,4,6-Trichloro-1,3,5-triazine (66 mg, 0.36 mmol, 0.2 eq) was dissolved in 1 mL of dry THF in argon atmosphere. Tetrakis(triphenylphosphine)palladium(0) (21 mg, 0.02 mmol, 1 mol%) was subsequently added in a counter flow of argon. To the reaction mixture the solution obtained in the previous step was added at room temperature, which resulted in an instantaneous color change to red. The reaction was refluxed until full conversion was observed by TLC. After the reaction mixture was cooled to room temperature 0.1 M HCl (10 mL) was added and it was extracted with diethyl ether (3x20 mL). The organic phase was washed with brine (40 mL) and dried over sodium sulfate. The solvent was evaporated and the raw product was purified by column chromatography (silica, light petrol:diethyl ether = 0-50%) and subsequent extraction of residual impurities by stirring in boiling *n*-hexane yielding 129 mg (47%) of an orange solid.

^{1}H-NMR (600 MHz, $CDCl_3$): δ= 7.53 (d, J = 9.0 Hz, 6H, Ar-$\underline{H}$), 6.57 (d, J = 9.0 Hz, 6H, Ar-$\underline{H}$), 3.30 (t, J = 7.8 Hz, 12H, -N-C$\underline{H}_2$-), 1.60 (m, 12H, -N-CH_2-C$\underline{H}_2$-), 1.36 (m, 12H, -C$\underline{H}_2$-CH_3), 0.97 (t, J = 7.4 Hz, 18H, -CH_2-C$\underline{H}_3$) ppm.

^{13}C-NMR (150 MHz, $CDCl_3$): δ= 160.5 (s, triazine), 149.7 (s, $\underline{C}$-N), 135.3 (d, -$\underline{C}$-C-C≡C), 111.2 (d, $\underline{C}$-C-N), 105.5 (s, $\underline{C}$-C≡C-triazine), 97.2 (s, -$\underline{C}$≡C-triazine), 87.3 (d, -C≡$\underline{C}$-triazine), 50.9 (t, -N-$\underline{C}H_2$-), 29.4 (t, -N-CH_2-$\underline{C}H_2$-), 20.4 (t, -$\underline{C}H_2$-CH_3), 14.1 (q, -CH_2-$\underline{C}H_3$) ppm.

HRMS (ESI): $[M + H]^+$ m/z calcd. for $C_{51}H_{67}N_6^+$ 763.5422; found 763.5412.

HRMS (ESI): $[M + 2H]^{2+}$ m/z calcd. for $C_{51}H_{68}N_6^{2+}$ 382.2747; found 382.2743.

T_m = 141 °C

Solvent Properties

The solvents were of the highest commercially available purity (Sigma-Aldrich, Acros organics, Alfa Aesar) and were used without further purification.

Supplementary Table 1. Solvents used for the experiments with their relevant macroscopic properties (n: refractive index; ε: static dielectric constant; $f(n^2)$: electronic polarizability function; $\Delta f = f(\varepsilon) - f(n^2)$ with $f(x) = 2(x-1)/(2x+1)$: Onsager polarity function; η: viscosity).

Solvent	n	ε	$f(n^2)$	Δf	η /cP	Abbrev.
2-Methylbutane	1.3537	1.84	0.3569	0.00	0.23	MeBu
Cyclohexane	1.4264	2.02	0.4081	0.00	0.98	CHX

Octane	1.3974	1.95	0.3885	0.00	0.55	OCT
Dodecane	1.4216	2.00	0.4050	0.00	1.51	DOD
Hexadecane	1.4210	2.01	0.4112	-0.01	2.8	HexDec
Paraffin oil	1.473	~2.0	0.4381	0.00	120	PAR
Vinyl acetate	1.3959	2.3	0.3874	0.08	0.42	VAc
Di-n-pentyl ether	1.4119	2.77	0.3984	0.14	1.10	DPE
Di-n-butyl ether	1.3992	3.08	0.3897	0.19	0.70	DBE
Diisopropyl ether	1.3681	3.88	0.3675	0.29	0.33	DiPrE
Benzene	1.5011	2.28	0.4552	0.00	0.65	BEN
Toluene	1.4969	2.38	0.4527	0.03	0.59	TOL
Diethyl ether	1.3524	4.34	0.3560	0.33	0.24	DEE
Butyl acetate	1.3940	5.01	0.3861	0.34	0.72	BuAc
Propyl acetate	1.3844	6.00	0.3793	0.39	0.59	PrAc
Ethyl acetate	1.3724	6.02	0.3707	0.40	0.45	EtAc
Tetrahydrofuran	1.4072	7.58	0.3952	0.42	0.55	THF
Dichloromethane	1.4242	8.93	0.4067	0.43	0.43	DCM
α,α,α-Trifluorotoluene	1.4140	9.40	0.3999	0.45		TrFTOL
Benzonitrile	1.5282	25.2	0.471	0.47	1.34	BZN
Dimethyl sulfoxide	1.4783	46.68	0.4414	0.53	2.24	DMSO
Acetone	1.3587	20.7	0.3606	0.57	0.32	AC
Propylene carbonate	1.4210	64.9	0.4046	0.57	2.5	PC
Acetonitrile	1.3441	37.5	0.3497	0.61	0.36	ACN
Butyronitrile	1.3838	20.3	0.3789	0.55	0.59	BN
Chloroform	1.4460	4.81	0.4211	0.30	0.57	$CHCl_3$

Steady-state Spectroscopy

Electronic absorption spectra were recorded on a Cary 50 spectrometer, whereas steady-state fluorescence spectra were recorded on a FluoroMax-4 (Jobin Yvon) and corrected using a set of secondary emissive standards.[7] Steady-state measurements were performed in 10 mm thick quartz cuvettes at a constant temperature of 20.0°C (293.15 K). Concentration of the samples was of the order of 10^{-6}-10^{-5} mol/L.

Steady-state Fourier-transform infrared spectra were acquired at 1 cm^{-1} resolution on a Bruker Tensor 27 FTIR spectrometer continuously purged with N_2 gas, averaging 64 scans at a constant temperature of 20.0°C (293.15 K). Samples were held between two 1 mm thick CaF_2 windows with 70 μm Teflon spacer defining the path length and mounted in a home-built brass sample cell connected to a recirculating chiller to control the sample temperature.

Two-photon Excited Fluorescence (TPEF) Spectroscopy

The optical scheme of the TPEF setup was similar to the one reported by Rebane and coworkers.[8] Part of the 800 nm output (450 μJ/pulse) of a 100 fs 1-kHz Ti:Sapphire amplified laser system (Spitfire, Spectra-Physics) pumped a collinear OPA (TOPAS-Prime, Light Conversion) equipped with a NirUVis mixing unit. It produced light pulses centered at different wavelengths tunable within 240-2600 nm using either the signal or idler beams or their higher harmonics. This tunable output was first routed through a series of silver and gold mirrors, then through a combination of a broadband zero-order half-wave plate and a vertically oriented Glan-Taylor polarizer which allows for adjustment of the excitation intensity. A lens was used to focus the excitation slightly into a 10 mm quartz cuvette containing the sample. The polarization of the excitation beam was set vertical in the laboratory frame. Two-photon excited fluorescence was collected at 90° by a slightly displaced 2-inch broadband dielectric concave mirror effective over the whole visible range of frequencies (400-750 nm), then focused onto the entrance of a grating spectrograph (Newport) and detected by a multipixel silicon avalanche photodiode (400 pixels, Hamamatsu). The signal was preamplified, processed with a gated boxcar-integrator and average module, digitized and recorded on a computer. The residual excitation was directed to a calibrated powermeter equipped with a thermal sensor (Thorlabs) to monitor beam intensity on a shot-to-shot basis. This was used both to correct for the laser intensity fluctuations and to correct automatically for the different TOPAS pulse energies when changing the excitation wavelength. Fluorescence was monitored around the spectral maximum (with 2 nm wide slit) using a 1-2 s integration time window, while the TOPAS scans the excitation wavelengths with an increment of 2 nm. Excitation pulse energies were of the order of few microjoules. The quadratic dependence of the fluorescence intensity on the excitation power was routinely checked at few selected wavelengths to ensure that no spurious one-photon-excited fluorescence was present. However, for the current work, there was no spectral overlap even between the lowest-wavelength range of the excitation beam for two-photon absorption (TPA) and highest-wavelength region of the stationary absorption spectrum. Therefore, no possible one-photon absorption could take place. Under these conditions, a two-photon fluorescence excitation spectrum is identical to a two-photon absorption spectrum and is referred to as such. Because spectra were recorded by scanning the wavelength of the TOPAS in order to obtain the full TPA spectrum, merging different wavelength regions was necessary. Spectra in each of the windows were obtained by averaging of 3-6 scans.

Time-correlated Single-photon Counting (TCSPC)

Fluorescence dynamics on the nanosecond timescale were measured using a time-correlated single photon counting setup described in detail previously.[9] Excitation was performed at 395 nm using ~60 ps pulses at 40 MHz produced by a laser diode (PicoQuant, LDH-P-C- 400B). The full width at half maximum (FWHM) of the instrument response function (IRF) was around 200 ps.

Transient Infrared (TRIR) Spectroscopy

Femtosecond TRIR spectra were obtained with the setup described in reference 10 and based on a Ti:Sapphire amplified system (Spectra Physics Solstice) producing 100 fs pulses at 800 nm at 1 kHz. Excitation was performed with 0.2-0.6 μJ pulses at 400 nm produced by frequency doubling a fraction of the amplifier output. The linearity of the signal amplitude with respect to excitation energy was checked before each experiment and proper adjustment of the pump intensity was made to ensure the maximum signal in the linear response regime. The polarization was controlled with a Glan-Laser polarizer and a zero-order half-wave plate, limiting the time resolution of the experiment to 300 fs. The pulses were focused on the sample to a spot of 350 µm resulting in an irradiance of 0.2-0.6 mJ/cm^2. Mid-IR probe pulses at around 3.5-6.1 µm were generated by difference frequency mixing of the output of an optical parametric amplifier (Light Conversion, TOPAS-C with NDFG module) that was pumped at 800 nm. The polarization of the IR beam was controlled using a wire-grid polarizer. Two horizontally polarized IR beams were produced with a CaF_2 wedge and focused onto the sample to a 140 µm diameter spot. One of the beams was overlapped with the pump beam, whereas the second one was used as a reference. Both IR beams were focused onto the entrance slit of an imaging spectrograph (Horiba, Triax 190, 150 lines/mm) equipped with a liquid nitrogen cooled 2x64 element MCT array (Infrared Systems Development), giving a resolution of ~3 cm^{-1}. The sample area and the detection system were placed in a box that was purged with water- and carbon dioxide-free air for at least one hour before each experiment as well as during the experiment. The average of 2000 signal shots was taken to collect one data point with the polarization of the pump at the magic angle to that of the IR pulse. This procedure was carried out 5-10 times. To provide a new sample solution for each shot, a flow cell as described in reference 11 was used. The absorbance at 400 nm was between 0.2-0.8 on a 200 µm optical pathlength. To avoid changes due to the evaporation of volatile cyclohexane, chloroform, diethyl ether, dichloromethane, THF and acetone during the experiment, sufficiently large sample reservoirs (~10 mL) were used. Given the bandwidth of the IR pulses (~400 nm), transient spectra recorded in up to 9 overlapping ca. 150-200 cm^{-1} spectral windows were merged to cover the 1650-2850 cm^{-1} spectral regions. All data sets were first compared to detect possible problems during the measurements. The spectra were then averaged and the different windows merged to obtain a single spectrum. The resulting spectra recorded were then compared, and regions without signal were inspected to identify the baseline. No tail matching had to be carried out. No significant sample degradation was observed throughout the experiment.

UV-vis Transient Absorption (UV-vis TA)

A detailed description of a femtosecond transient absorption applying referenced detection using two spectrographs is presented elsewhere.[12] The visible transient absorption setup used in this work was based on this general scheme. Excitation was performed using 400 nm pulses generated by frequency doubling part of the output of a 1 kHz Ti:Sapphire

amplified system (Spectra Physics, Solstice Ace). The transient absorption signal was checked prior to the experiment to scale linearly with the pump energy. The polarization of the pump pulses was set to magic angle relative to the white-light pulses. In order to compensate for pump beam divergence and/or delay-line misalignment, the fs-TA dynamics were corrected by comparing with a calibration sample (perylene in DMSO) measured on the TA and the TCSPC setups. Probing was achieved using white light pulses generated by focusing the 800 nm pulses of the Ti:Sapphire amplified system in a CaF_2 plate. The experimental setup was the same as that described earlier,[13] except that all lenses, after white light generation, were replaced by spherical mirrors to prevent chromatic aberration. The pixel to wavelength conversion was achieved using a standard containing rare earth metals (NIST 2065) which shows narrow bands from the UV to the NIR. All transient absorption spectra were corrected for background signals showing up before time zero (e. g. spontaneous emission). Furthermore, the spectra were corrected for the dispersion due to the optical chirp using the optical Kerr effect.[14] The samples were measured in 1 mm quartz cuvettes (Starna, model 1GS/Q/1) and bubbled with nitrogen gas during the measurements giving a wavelength dependent IRF of about 80-350 fs (fwhm). The absorbance of the sample at the excitation wavelength was 0.2-0.5 (at 400 nm) on 1 mm path length. The absorption spectra of all samples before and after the transient absorption experiments showed no signs of degradation.

Near-infrared (NIR) Transient Absorption (NIR TA)

Near-infrared transient absorption setup was based on the same scheme as the UV-vis TA, used the same laser source and identical experimental procedures were applied.[12] The white probe light was generated by focusing 800 nm pulses in a YAG crystal. To balance the intensity of the white light the high intensity 800 nm light was removed by a beam stop after generation as well as by a 1 mm cuvette containing IR140 in DMSO. The probe light was then separated into a reference and a sample beam by a reflective metallic neutral density filter. After passing the sample the beam was dispersed by a home-built prism spectrometer and the intensity recorded with an InGaAs detector. To further suppress the high intensity around 800 nm, apodizing neutral density filters were placed directly before both detectors.

For merging the spectra recorded with the UV-vis and NIR TA setups, the signal in the overlap region between 690 and 740 nm was compared and one of the two datasets was multiplied with a constant factor accounting for the difference in pump power. The comparison of the kinetics recorded in the overlap region additionally serves as quality control directly revealing erroneous kinetics, e.g. due to deviations from the magic angle or poor alignment of the delay stage.

Fluorescence Upconversion Spectroscopy (FLUPS)

Fast (fs to hundreds of ps) fluorescence dynamics were investigated by broadband fluorescence up-conversion spectroscopy (FLUPS) with a setup similar to that reported in references [17–19]. In brief, excitation was performed with 100 fs pulses at 400 nm generated by frequency doubling part of the output of a standard 1 kHz Ti:Sapphire amplified system. The pump intensity on the sample was below 1 mJ/cm^2. The gate pulses were at 1340 nm and were produced by an optical parametric amplifier (TOPAS-C, Light Conversion). Detection of the up-converted spectra was performed with a CCD camera (Andor, DV420ABU). The FWHM of the cross correlation of the gate with the solvent Raman signal was approximately 170 fs. Corrected time-resolved emission spectra were obtained by calibration with secondary

emissive standards as described in reference 20. Time-resolved emission spectra were recorded in two sequential measurements: a linear time-step range from -2 to 2 ps and a logarithmic time-grid covering the remainder of the time window. The temporal chirp was determined measuring the instantaneous response of BBOT (Radiant Dyes, used as received) in all of the used solvents. The experimental raw data were henceforth corrected for the temporal chirp and then transferred into corrected spectra vs. wavelength or wavenumber according to reference 20.

Quantum-Chemical Calculations

DFT calculations at B3LYP/6-311+G(d,p) or CAM-B3LYP/6-311+G(d,p) levels of theory were used for **D** as implemented in Gaussian 09 (Rev. D.01)[21]. PCM model was used to account for the solvent reaction field.

Global Target Analysis of Spectrotemporal Data

The transient data were analyzed globally using a target model consisting of a series of successive exponential steps, A→B→C→…[22,23] This analysis yields the species-associate difference absorption spectra (SADS) of A, B, C, …, and the time constants of the different steps. In many cases, A, B, C cannot be assigned to a precise species or state but rather correspond to the same species/state at different stages of its relaxation and/or comprises contribution of several species/state if a more complex kinetics than just a sequential model are present. In those cases, it is preferable to speak about evolution-associated difference absorption spectra (EADS) rather than SADS and about timescales rather than time constants.

SUPPLEMENTARY DISCUSSION

Steady-state Electronic Spectroscopy

Steady-state electronic absorption (top) and fluorescence (bottom) spectra of **O** (left) and **Q** (right) in a comprehensive set of 17 solvents with gradually increasing Onsager dipolar reorientation function, Δf, are shown in **Supplementary Figure 1**. Very strong charge-transfer character of these molecules is evident from the solvent dependence of the absorption spectra. The position of the band maximum varies within ~2000 cm^{-1} (397-430 nm) for **O** and ~2800 cm^{-1} (392-420 nm) for **Q**. Such a strong solvent dependence is stunning, especially for the octupolar system. Similar behaviour was recorded with analogous **Q** and **O** systems, where the alkyne π-bridge is changed for the alkene trans-C=C fragment (not shown).[24] The double-bonded systems are less rigid and can adopt a number of more polar conformations that are inaccessible to triple-bonded ones but their solvatochromic shifts are smaller (~1725 cm^{-1} for **Q** and ~1900 cm^{-1} for **O**) compared to the alkynyl-bridged analogues. Therefore, molecular flexibility due to conformational degrees of freedom is not at the origin of this effect.

For **O** and **Q**, no good correlation exists between the maximum of the absorption band ($\tilde{\nu}_a$) and either Onsager electronic, $f(n^2)$, or dipolar, Δf, polarizability functions taken alone. However, it correlates well when both are taken together in the form:

$$\tilde{\nu}_\mathrm{a} = \tilde{\nu}_\mathrm{a}^0 - \mathrm{A}f(n^2) - \mathrm{B}\Delta f \qquad (1)$$

where $\tilde{\nu}_\mathrm{a}^0$ is the band maximum in the gas phase and the coefficients A and B are determined from multilinear regression (**Supplementary Figure 2a, Supplementary Table 2**). The excellent linear dependence is also obtained when the absorption solvatochromism is correlated with the Kamlet-Taft π^* parameter[25] that encompasses both dipolar and dispersive interactions (**Supplementary Figure 2b**). Moreover, the slope of such dependence for both multipolar systems is essentially identical (**Supplementary Table 3**).

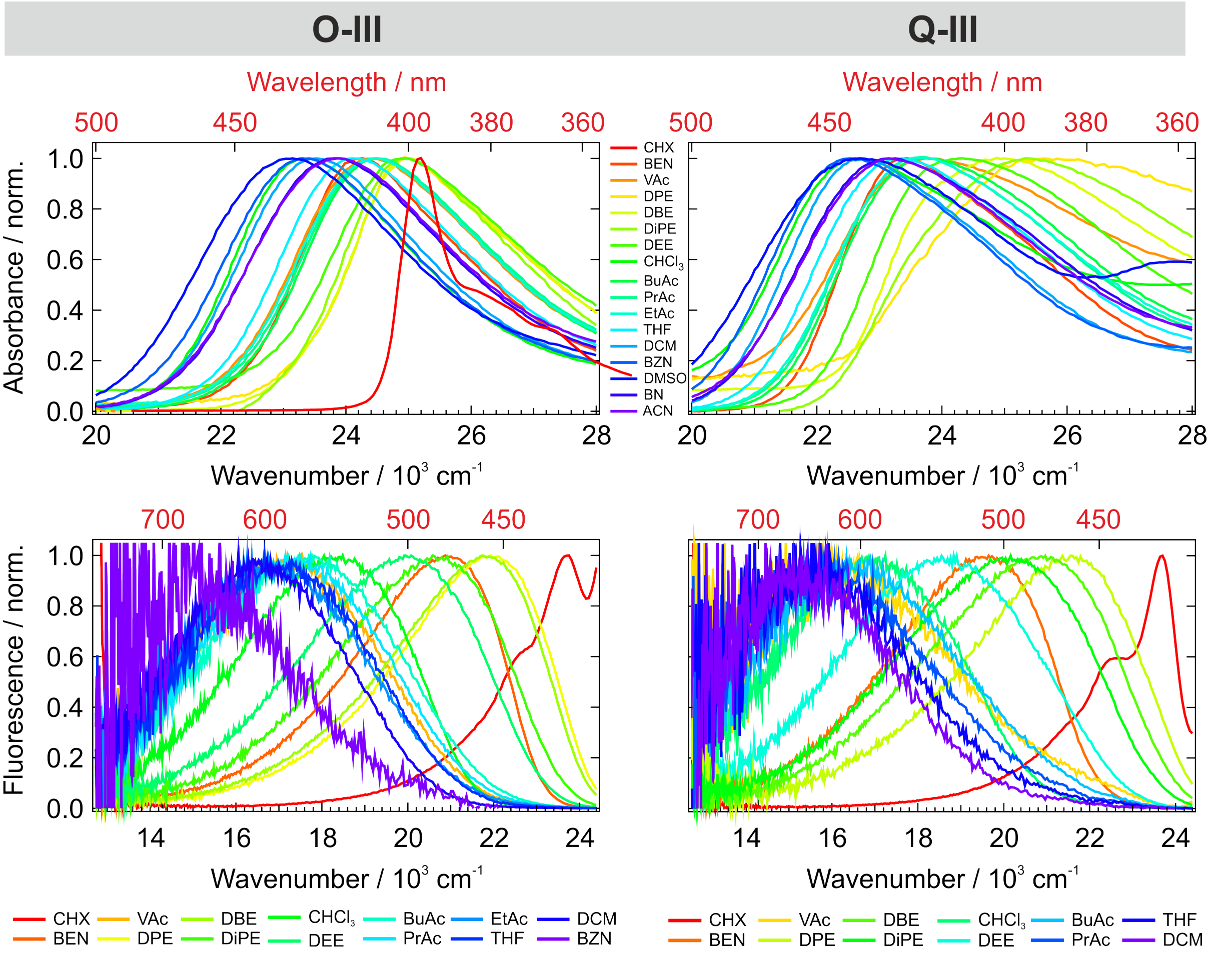

Supplementary Figure 1. Normalized steady-state electronic absorption (top) and emission (bottom) spectra of the octupolar (left) and quadrupolar (right) molecules. Full data sets include 17 solvents of increasing polarity, but not all of the fluorescence spectra are shown for visual clarity.

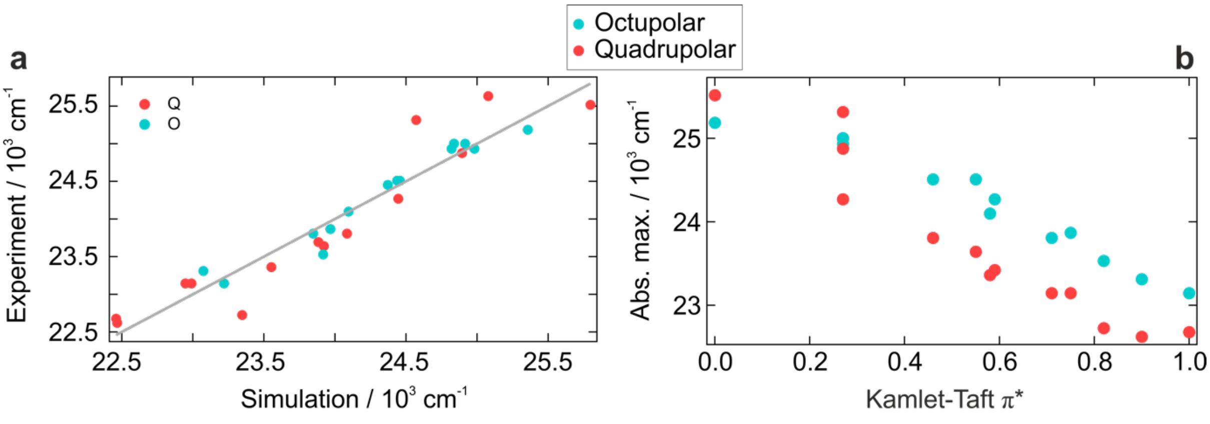

Supplementary Figure 2. Steady-state electronic absorption solvatochromism of **O** (cerulean) and **Q** (red). **a.** Comparison between experimental and simulated data taking into account both electronic and dipolar reorientation polarizability according to Eq. (1). **b.** Lowest-energy absorption peak maximum versus Kamlet-Taft π^* parameter.

It points out that, for these multipolar dyes, there is a substantial dipolar contribution to their solvatochromism (**Supplementary Table 4**). This is different from other examples of

quadrupolar molecules that we have investigated before, where electronic polarizability was the only factor determining the spectral position of the very weakly solvatochromic absorption band.[10,26–28] Moreover, the absorption band spectral shifts are larger than those for purely dipolar system **D** (**Supplementary Figure 3**, **Supplementary Table 3**).

Supplementary Table 2. Multilinear fit parameters extracted from the absorption solvatochromism data for **O**, **Q** and **D** fitted to Eq. (1).

System	$\tilde{\nu}_a^0$ / cm^{-1}	A / cm^{-1}	B / cm^{-1}
**O**	29930	11227	3333
**Q**	30062	10487	5646
**D**	28270	12048	1450

Supplementary Table 3. Linear fit parameters extracted from the absorption solvatochromism dependence on Kamlet-Taft π* for the octupolar, quadrupolar and dipolar dyes. For **D**, additional fit parameters are shown for its dependence on Δf (color-shaded).

System	Slope / cm^{-1}	Intercept / cm^{-1}
**O**	2570±120	25706±77
**Q**	2420±230	24870±130
**D**	1550±70	24405±38
**D** (Δf)	2100±190	24390±74

Supplementary Table 4. Individual contributions to the observed spectral shifts from the electronic polarizability ($f(n^2)$) and dipolar reorientation functions (Δf) for **O** and **Q** with respect to the gas-phase values according to Eq. (1). The values of the polarizability functions are also shown.

Solvent	$f(n^2)$	Δf	**O**		**Q**	
			$f(n^2)$ / cm^{-1}	Δf / cm^{-1}	$f(n^2)$ / cm^{-1}	Δf / cm^{-1}
Cyclohexane	0.4081	-0.0026	4582	-9	4280	-15
Di-n-pentyl ether	0.3984	0.1429	4473	476	4178	807
Di-n-butyl ether	0.3897	0.1917	4375	639	4087	1082
Diisopropyl ether	0.3675	0.2900	4126	966	3854	1637
Diethyl ether	0.3560	0.3338	3997	1112	3733	1884
Butyl acetate	0.3861	0.3417	4335	1139	4049	1929
Propyl acetate	0.3793	0.3900	4259	1300	3978	2202
Ethyl acetate	0.3707	0.3992	4162	1330	3887	2254
Tetrahydrofuran	0.3952	0.4192	4437	1397	4144	2367
Dichloromethane	0.4067	0.4342	4566	1447	4265	2451
Benzonitrile	0.4710	0.4706	5288	1568	4939	2657
Dimethyl sulfoxide	0.4414	0.5268	4956	1756	4629	2974
Butyronitrile	0.3789	0.5490	4254	1830	3973	3099
Acetonitrile	0.3497	0.6108	3926	2036	3667	3448

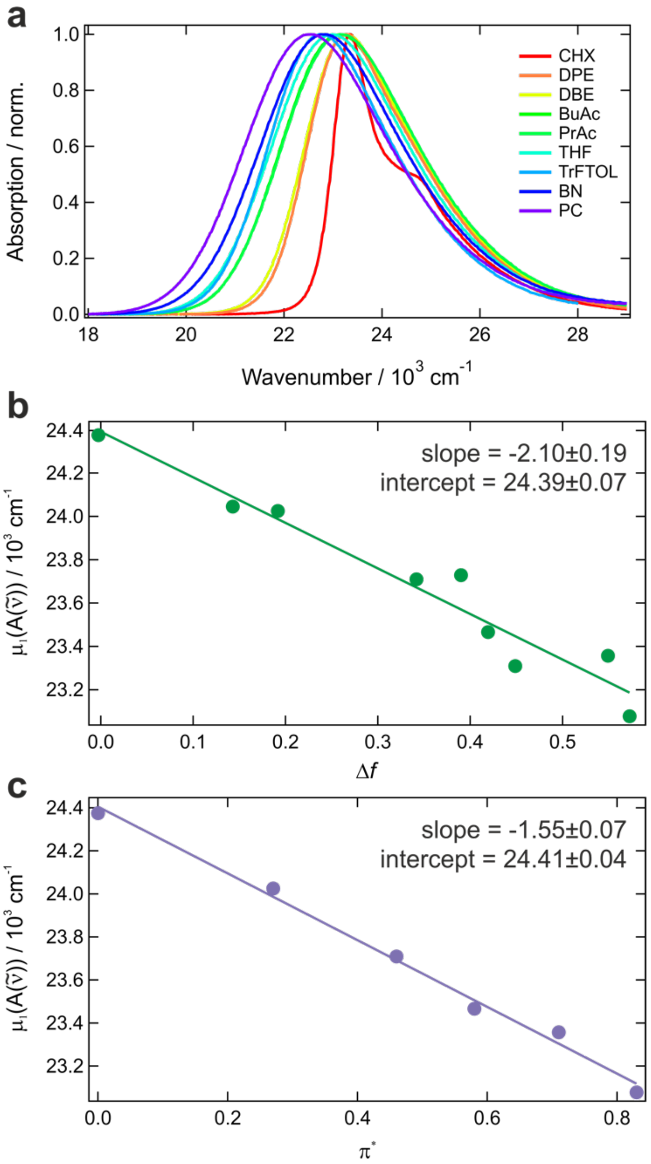

Supplementary Figure 3. Steady-state electronic absorption solvatochromism of **D**. **a.** Absorption spectra recorded in several solvents with similar values of $f(n^2)$ and varying Δf to minimize the contribution of the electronic polarizability. **b.** Best linear fit of the first moment of the absorption spectrum versus Δf. **c.** Best linear fit of the first moment of the absorption spectrum versus Kamlet-Taft π^* parameter (only the solvents for which π^* parameter is known are plotted).

What is the origin of such a dramatic solvatochromism if the ground state is symmetric and multipolar? In case of **Q**, one could consider solvation of a quadrupole by a continuum of dipolar solvent molecules. This is a natural extension of the traditional Lippert-Mataga model for solvation of a dipole with the same set of assumptions. In this case, the solvent's orientation polarization is described by

$$\Delta E_{\rm or} = -\frac{Q_{\rm g}(Q_{\rm e} - Q_{\rm g})}{2a^5}\Delta f \qquad (2)$$

where Q_g and Q_e are molecule's quadrupolar moments in electronic ground and excited states respectively and a is the Onsager cavity radius. The effective molecular quadrupolar moment in the S_0 state, Q_g, can be calculated from the traceless quadrupole moment tensor $\bar{Q}$ obtained from the quantum-chemical calculation by

$$Q_g = \frac{3}{2}\bar{Q}:\bar{Q} \tag{3}$$

The Onsager cavity radius could be estimated either from the effective radius obtained by computing Van der Waals volume of the molecule or simply by taking half of the value of distance between the two extremities. In either case, since the shape of the molecule is far from spherical, it provides only a rough estimate for this model and, in principle, empirical shape correction factors as well as the dipole position correction factor could be used.[29] There is no reliable experimental way to evaluate the quadrupolar moment in the excited state, Q_e but we can estimate what change of quadrupolar moment is required to explain the observed solvatochromism according to Eq. (2) and how sensitive its value is with respect to Q_g and a (**Supplementary Table 5**).

Supplementary Table 5. Calculation of the quadrupolar moment increase ($\Delta Q = Q_e - Q_g$) upon photoexcitation assuming that the observed absorption solvatochromism arises purely from the orientational solvation of the solute quadrupolar moment by solvent dipoles according to Eq. (2) based on the observed solvatochromic shift in highly polar acetonitrile. The best estimates of the cavity radius and ground-state quadrupolar moment are shaded in green.

ΔQ (in DÅ) for different a, if Q_g = 200 DÅ			
**6 Å**	**7 Å**	**8 Å**	**9 Å**
60	130	250	455
ΔQ (in DÅ) for different Q_g, if a = 8 Å			
**100 DÅ**	**150 DÅ**	**200 DÅ**	**250 DÅ**
505	337	250	202

The best estimate of Q_g for Q is about 150-200 DÅ according to DFT calculations. The final result does not depend very dramatically on the precise value of Q_g used (**Supplementary Table 5**). It is more sensitive to the value of the cavity radius, which is less certain. **Supplementary Table 5** demonstrates that regardless of the exact values used, the overall quadrupole moment should increase by factor of 2-3 in order to explain the observed spectral shifts. If connected to a simple model of a quadrupole as an arrangement of two oppositely pointing dipoles $\vec{\mu}$ with angle α and distance d between their centers, then an increase of the ground-state quadrupolar moment by a factor of 2 leads to an increase of the dipole moment of each arm by more than 32 D, which is unrealistically large (it is at least factor of 3 larger than the dipole moment change in a single-arm molecule).

However, although described and used in several cases[29,30], the major pitfall of this model is the underlying assumption of the point quadrupole, which fails spectacularly in the current case. The distance dependence of such a quadrupole-dipole interaction is steep ($\sim a^{-5}$) meaning that only the closest solvent molecules should play substantial role in solvation of a quadrupole. **Q** has long protruding charge-transfer arms and is substantially larger than the surrounding solvent molecules. The solvent dipoles in the first few solvation shells don't feel the quadrupolar field of the solute but rather they sense the dipolar field of the individual branches or even smaller subunits. Therefore, a crude solute point-quadrupole approximation breaks down in this case and the substantial absorption solvatochromism can be ascribed to the local dipole-dipole interactions between the solute extended charge-transfer arms and the solvent dipoles. This is the only way to explain the comparative magnitude of the solvatochromic shifts for octupolar **O**, which otherwise should imply a very exotic octupole-dipole interaction (with distance dependence $\sim a^{-7}$) of an exceptional magnitude.

Therefore, it is the strong dipolar character of the individual **D-A** arms paired with their bulkiness that lead to a breakdown of the common approach based on the point dipole/multipole approximation. In polar media, solvent molecules in the first few solvation shells interact with the local dipoles in the individual arms rather than with a multipolar charge distribution set by the entire molecular $\mathbf{A(-D)_n}$ system. Only those solvent dipoles which are more than several solvation shells away from $\mathbf{A(-D)_n}$ could sense its multipole, but they play a very minor role in the solvation of these structures because *i)* solvent dipole-solute multipole interactions decay very steeply with distance ($\sim r^{-5}$ for dipole-quadrupole and $\sim r^{-7}$ for dipole-octupole interactions) and *ii)* the dielectric screening becomes progressively important in more polar solvents leading to a rapid decay of the electrostatic interaction.

Supplementary Figure 1 (bottom) demonstrates even more spectacular emission solvatochromism of **O** and **Q** that covers the whole visible range from blue in apolar medium to red in the most polar solvents. The band maximum varies within 420-690 nm (~9220 cm^{-1}) for **O** and 420-650 nm (~8300 cm^{-1}) for **Q**. In non-polar environment, fluorescence spectra of both molecules are narrow with a ~1100 cm^{-1} vibronic progression. They redshift and broaden as solvent polarity increases (**Supplementary Figure 4a**). The fluorescence quantum yield in apolar solvents is ~48 % for **O** and ~28 % for **Q** but goes down dramatically to <1 % in highly polar ones (**Supplementary Table 6**). The octupolar dye has a notably higher fluorescence efficiency in all solvents.

Supplementary Table 6. Fluorescence quantum yields (%) of the multipolar dyes in solvents of increasing polarity.

Solvent	O	Q
Cyclohexane	48	28
Benzene	35	21
Vinyl acetate	5	2
Di-n-pentyl ether	26	12
Di-n-butyl ether	29	16
Diisopropyl ether	15	12
Chloroform	10	4
Diethyl ether	14	10
Butyl acetate	7	4
Propyl acetate	6	2

Ethyl acetate	4	1
Tetrahydrofuran	5	1
Dichloromethane	4	1
Benzonitrile	0.8	0.3
Dimethyl sulfoxide	<0.1	0.2
Butyronitrile	0.4	0.4
Acetonitrile	<0.1	<0.1

Supplementary Figure 4b demonstrates that the emission transition dipole moment (TDM) decreases significantly in a non-linear fashion with increasing polarity of the environment as the $S_1 \rightarrow S_0$ energy gap goes down. Even in the apolar environment, where the largest emission TDM of ~4 D is observed, this value is significantly lower than the corresponding absorption TDM of 7.3 D. Substantial curvature of the plot of the emission transition moment versus the energy gap is evident but cannot be fully appreciated because it is not possible to determine transition moments in the 3-4 most polar solvents due to the ultrafast fluorescence decay and negligible fluorescence quantum yield. Therefore, the TDM is even lower in these solvents. These transition dipole values and trend indicate that the nature of the relaxed excited state is not the same as the ground state of the molecules and it is continuously changing to less emissive from apolar to polar solvents.

From **Supplementary Figure 4c** it is clear that the non-radiative rate constant does not follow the energy-gap law for radiationless transitions for **Q** as it stays approximately constant in the least polar solvents, and increases by a factor of 2-3 in more polar ones. Octupolar chromophore **O** exhibits similar but somewhat less pronounced behavior.

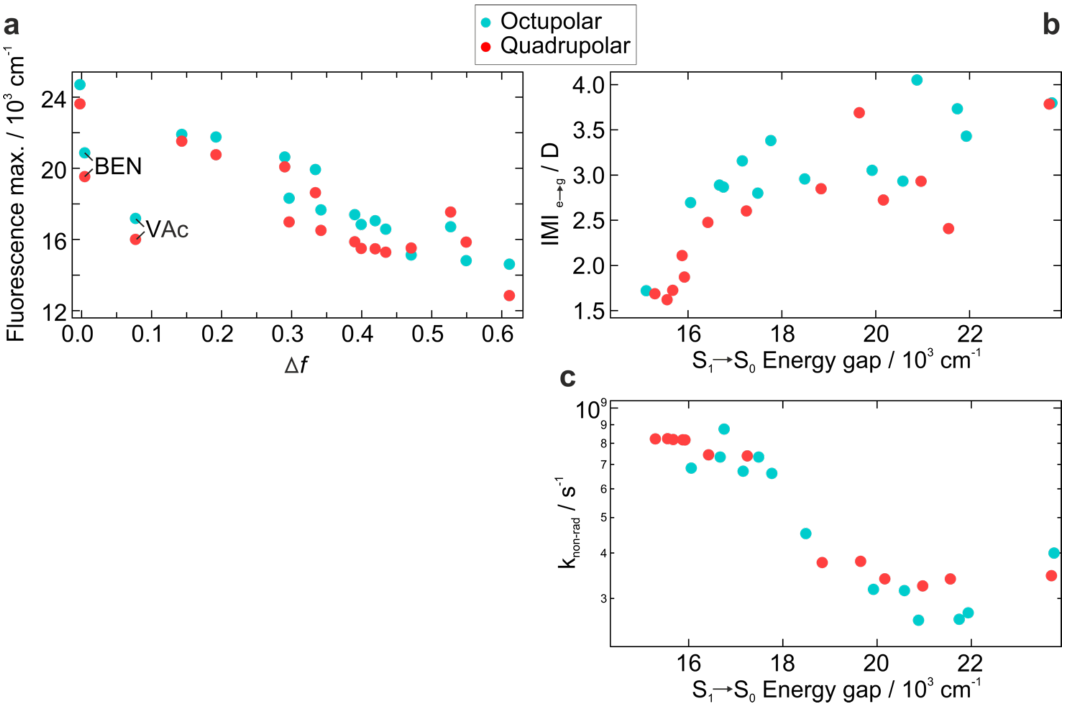

Supplementary Figure 4. a. Dependence of the emission band maximum on the Onsager dipolar polarizability function. Benzene and vinyl acetate appear as prominent outliers due to their substantial quadrupolar character not accounted for by Δf.[27] **b.** Emission transition dipole moment as a function of the $S_1 \rightarrow S_0$ energy gap. **c.** Dependence of the non-radiative rate constant on the $S_1 \rightarrow S_0$ energy gap.

Steady-state Fourier-transform Infrared (FTIR) Spectroscopy

The FTIR spectrum of **D** shows a distinct single resonance around 2190 cm^{-1}, in the spectral region corresponding to the alkynyl C≡C stretch vibration. This resonance exhibits vibrational solvatochromism as its frequency downshifts when the solvent reaction field is increased mainly due to vibrational Stark effect (**Supplementary Figure 5**).[31,32] For example, in the apolar cyclohexane it peaks at 2193.5 cm^{-1}, whereas in the quadrupolar aromatic toluene its value is 2190.2 cm^{-1} and 2182.0 cm^{-1} in chloroform.

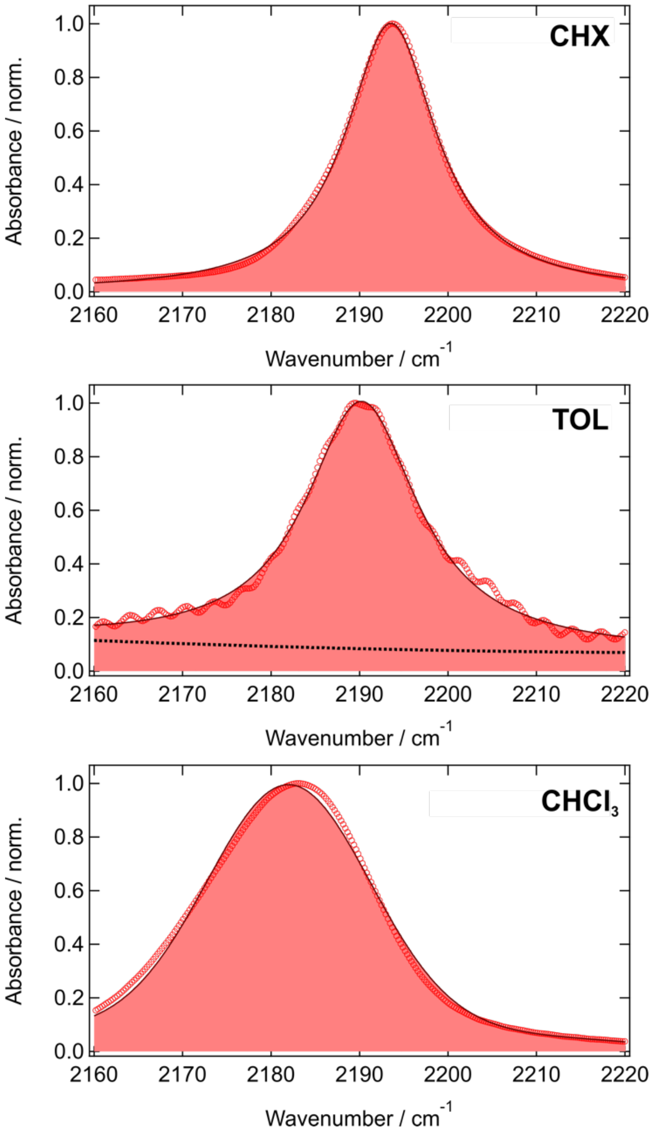

Supplementary Figure 5. FTIR spectra of **D** in several representative solvents of increasing polarity. Red markers: experimental data points; red fill: Lorentzian/Gaussian fit to the band; dashed black line: background; solid line: total fit.

The FTIR spectra of **O** and **Q** compared to **D** in chloroform are shown in **Supplementary Figure 6**. Since in **Q** the angle between the arms is 120°, the selection rule forbidding the symmetric stretch vibration band in the IR spectrum is partially lifted and it

appears as a weak band at 2178.7 cm^{-1}, whereas the intense band for antisymmeric stretch is at 2190.1 cm^{-1}. (**Supplementary Figure 6**, middle panel). The integral intensity of the symmetric stretch is about 7% of the antisymmetric one. In the local mode picture, the coupling between the two C≡C stretches amounts thus to 5.7 cm^{-1}. **O** has three C≡C stretch vibrations, one of which (totally symmetric A_1') is strictly forbidden, whereas the other two (E' symmetry) appear split at 2179.5 and 2194 cm^{-1} giving thus the value of 4.8 cm^{-1} for vibrational coupling between the individual C≡C stretches in each arm. The value of the vibrational coupling between individual branches in the S_0 state is ~5 cm^{-1} for **Q** and **O**. On the other hand, DFT calculations ignore this coupling and predict the stretches to be essentially degenerate (~0.1 cm^{-1} splitting).

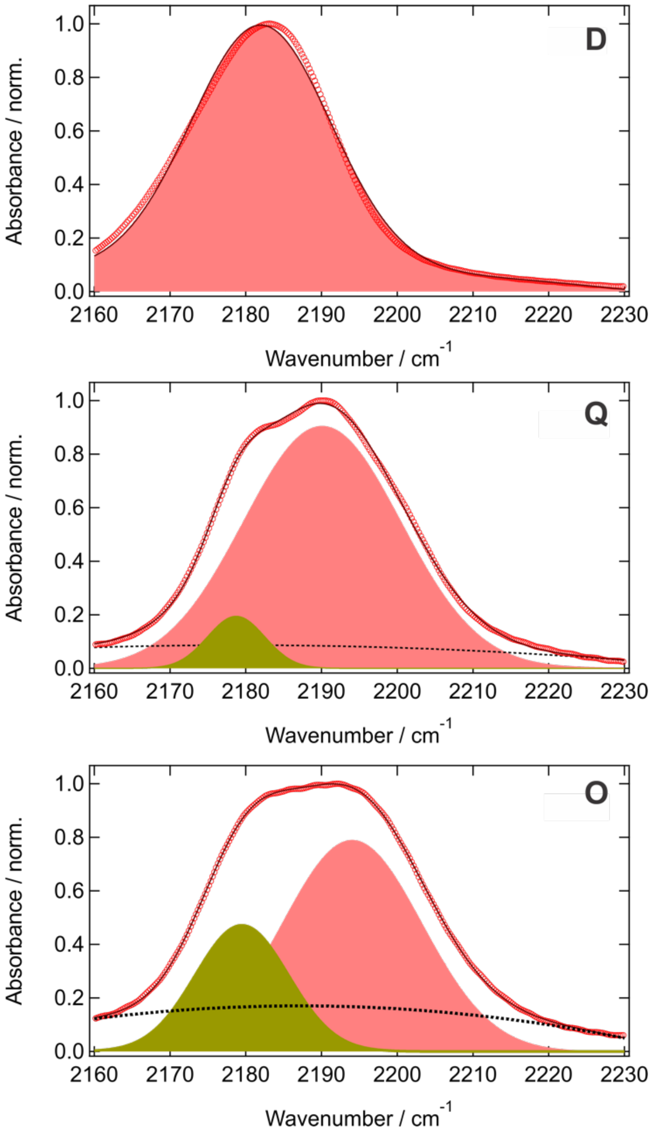

Supplementary Figure 6. FTIR spectra of **D**, **Q** and **O** in chloroform. Red markers: experimental data points; red/dijon fill: Gaussian fits to the bands; dashed black line: background; solid line: total fit.

TRIR

Electronic excitonic transient absorption of O and Q

The weak coupling and strong charge shifts in the excited state cause exciton localization even in the weakest polar solvents used in this study, such as the quadrupolar benzene, or the low-polar ethers. The initially present excitonic band disappears and confirms the prediction of the Ivanov model. **Supplementary Figure 7** visualizes the decay of the excitonic band in several solvents of increasing polarity by showing evolution-associated difference TRIR spectra obtained from global target analysis of early spectra within the first 10-20 ps. At this point, we focus only on the dynamics of the electronic band and disregard vibrational bands seen at later times once the excitonic transition has vanished. The dynamics are essentially identical for both octupolar and quadrupolar compounds and follow solvation dynamics (**Supplementary Table 7**).

Additionally, the global analysis was performed on the 2250-2850 cm^{-1} spectral region alone where only the excitonic electronic ESA signal is present, and no vibrational features have resonances. This provides a handle on pure excitonic band dynamics without interference from dynamics of vibrations at later times. We have found the same dynamics as presented in **Supplementary Figure 7** from the entire observation window. This is expected because the amplitude of the electronic absorption overwhelms that of the vibrational bands and the global analysis is mostly sensitive to the dynamics of the excitonic band. It also demonstrates that, in order to study the full range of vibrational dynamics, it is necessary to get rid of the electronic resonance contribution.

The symmetry-preserved delocalized state is observed only in a truly apolar medium that contains no polar bonds or conjugated π-systems. **Supplementary Figure 14** shows that the only spectral dynamics happening during the first few ps in cyclohexane is a very small intensity redistribution: the low-frequency edge of the spectrum loses ~10 % of the intensity, whereas the high-frequency edge of our observation window gains ~5 % of the intensity (A→B transformation). Such intensity redistribution signifies narrowing from the low-frequency side of the excitonic band and happens on ~4 ps time scale. It is due to the vibrational cooling (VC) of the low-frequency modes populated after intramolecular vibrational energy redistribution process (IVR) dissipates excess energy delivered with 400 nm visible pump photons.

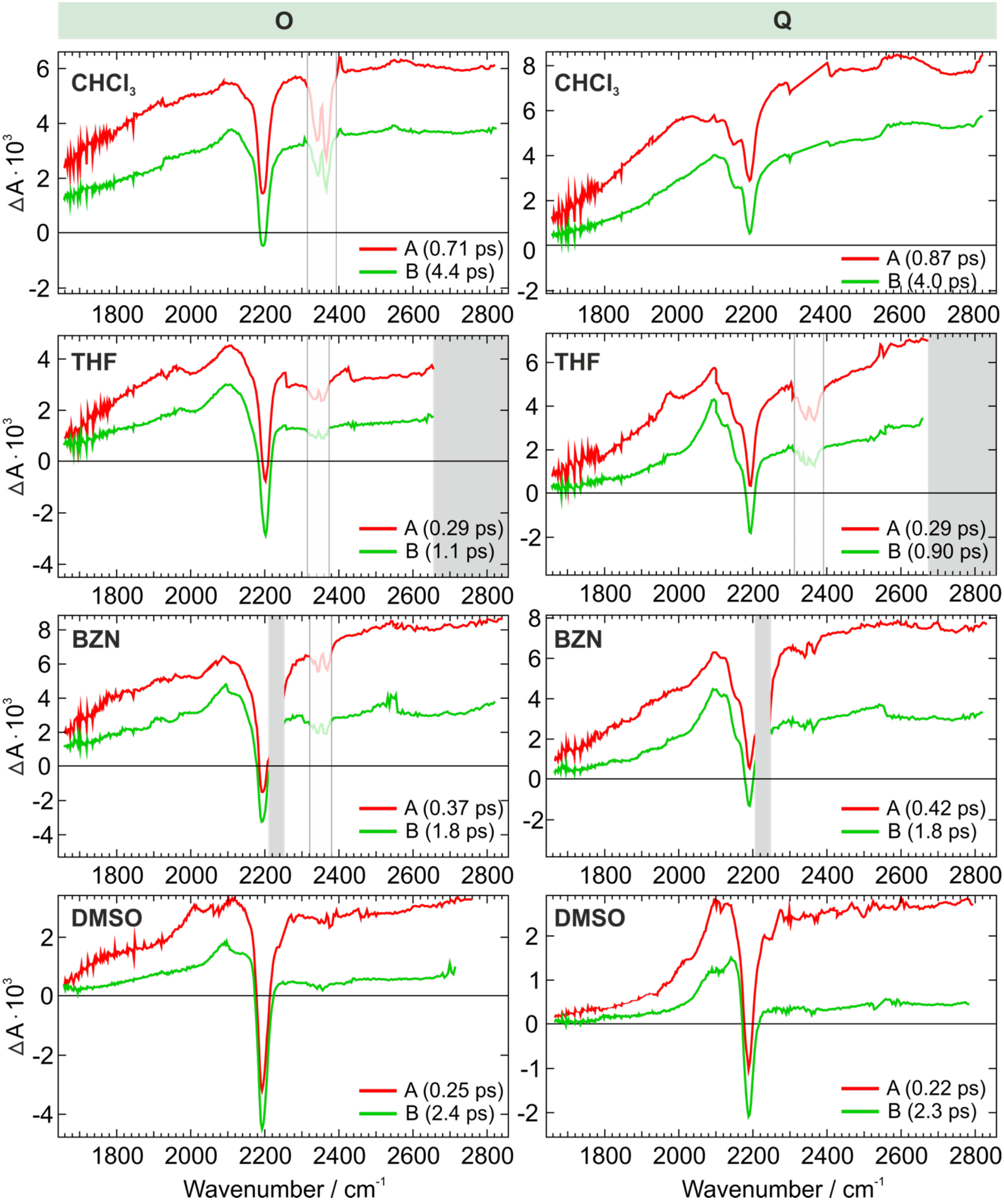

Supplementary Figure 7. Evolution-associated difference spectra obtained from global target analysis (A→B→C→(D)→) of early TRIR spectra of **O** and **Q** in several representative solvents of increasing polarity. The region near 2350 cm^{-1} is shaded to cover interferences caused by incomplete removal of atmospheric CO_2.

Supplementary Table 7. Symmetry-breaking timescales recovered from the global analysis of the excitonic $S_2 \leftarrow S_1$ absorption band decay compared to the literature solvation timescales.

	$CHCl_3$	THF	BZN	DMSO
**O**	0.71 ps<br>4.4 ps	0.29 ps<br>1.1 ps	0.37 ps<br>1.8 ps	0.25 ps<br>2.4 ps
**Q**	0.87 ps<br>4.0 ps	0.29 ps<br>0.90 ps	0.42 ps<br>1.8 ps	0.22 ps<br>2.3 ps
Literature[38]	0.29 ps (36 %)<br>4.2 ps (64 %)<br>**<τ> = 2.8 ps**	0.23 ps (45 %)<br>1.52 ps (55 %)<br>**<τ> = 0.94 ps**	0.36 ps (38 %)<br>5.3 ps (53 %)<br>25 ps (9 %)<br>**<τ> = 5.1 ps**	0.21 ps (50 %)<br>2.3 ps (41 %)<br>11 ps (9 %)<br>**<τ> = 0.94 ps**

Vibrational dynamics of model D compound

A photochemical alkyne-allene isomerization of the π-linker occurs with the model dipolar **D** compound.[1] The rate of the reaction is largely governed by solvent viscosity as expected in case of large-amplitude motion. However, since the reaction is accompanied by electron transfer, the rate is also substantially modulated by the dielectric properties of the environment. **Supplementary Figure 8** presents reaction rate (τ_{iso}) dependence for **D** in an augmented set of 14 weakly to strongly polar solvents along with the fit to the expected viscosity (η) dependence of the following form:

$$\tau_{\text{iso}} = A\eta^{\alpha} \tag{4}$$

where A and α ($0 < \alpha < 1$) are constants for a given compound.

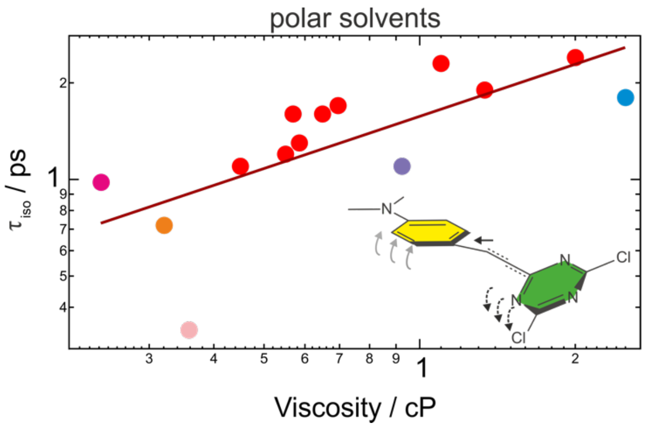

Supplementary Figure 8. Viscosity dependence of the time constant of alkyne-allene isomerization of **D** in polar solvents along with the best fit according to Eq. (4). The inset illustrates the large-amplitude motion associated with the process.

Whereas the model in general describes the viscosity dependence of the rate constant, the scatter of the values is apparent and originates from the polarity modulation of the electron-transfer rate that affects the isomerization. In solvents of similar viscosities, the reaction proceeds faster in more polar media. For example, three low viscous solvents of differing

polarities (diethyl ether – magenta, acetone – orange and acetonitrile – pink) exhibit a large modulation of the reaction rate. The strongly polar dimethyl formamide (flint) and propylene carbonate (cerulean) appear to accelerate reaction as well. Therefore, the great advantage of apolar environment for this reaction is the elimination of the polarity effect.

The spectral evolution of TRIR spectra of this compound in the apolar cyclohexane is shown in **Supplementary Figure 9a** and the results of the global target analysis are shown in **Supplementary Figure 9b**. The ground-state bleach is centered around 2194 cm^{-1} and, at the earliest time, an intense excited-state absorption band peaks at 2071 cm^{-1} with a prominent asymmetric tail on the low-frequency side stretching up to where allene absorbs. The molecules exist as a distribution of conformations with various dihedral angles around the triple bond and the frequency of the bridge is a sensitive marker for the angular distribution. Since the planar molecules have the highest IR frequency and the twisted allene photoproduct has the lowest, this asymmetric tail reports on the distribution of the dihedral angles initially centered on the planar configurations but shifting to the perpendicular ones as isomerization proceeds (**Supplementary Figure 9b**). This band rapidly transforms into a new one with a maximum at 1920 cm^{-1} and a tail on the high-frequency side stretching up to the position of the initial ESA peak. This transformation is characterized with non-exponential kinetics which could be fitted well with a sum of two exponential functions (1.4 and 6.6 ps for CHX) (**Supplementary Figure 10b**). The GSB amplitude remains constant during all these changes pointing out that all processes happen on the excited-state potential energy surface. These changes are associated with the transformation of the initial linear alkyne-bridged molecule to a twisted and bent allene-bridged one as we reported before.[1] Afterwards, 78 % of the molecules relax to the ground state, whereas 22 % find their way to the triplet manifold characterized by the symmetric absorption peak at 2066 cm^{-1} highlighting the alkyne spacer IR absorption in the T_1 state. The singlet excited-state lifetime in apolar environment is ~1.8 ns.

The characteristic lineshapes of the alkyne-state and allene-state excited-state IR spectra, as well as prominently non-exponential transformation from one to the other, point out that the molecular ensemble exists as a distribution of geometries characterized by varying donor-acceptor dihedral angles projected from the ground state. The triplet state is geometrically less flexible and exists as a fully planar linear alkyne as evidenced by the symmetric lineshape of its absorption peak. The alkyne-allene transformation can be viewed as an effective shift of the ensemble towards an allenic-type structure being accompanied by an intramolecular charge separation.

TRIR experiments were carried out in a series of 6 apolar aliphatic hydrocarbons of varying viscosity. The spectral behavior is identical in all aspects for all the investigated solvents, except for the quantitative dynamics. The reaction slows down considerably upon increasing viscosity as expected from Eq. (4). Both time constants used to approximate the non-exponential reaction dynamics change in unison (**Supplementary Figure 10a**) and, according to the amplitudes of EADS A, B and C both time constants have approximately the same contributions. Therefore, we estimate the effective reaction time constant as an average of the two, and this effective rate is shown in **Supplementary Figure 10b** along with the best fit of eq. (4). It is clear that there is an excellent correspondence to the expected power-law behavior and the scatter of the values around the best fit line present in **Supplementary Figure 8** for polar solvents is completely removed (**Supplementary Figure 11**). The results of the fits of Eq. (4) to the data both for apolar and polar solvents are presented in **Supplementary Table 8**.

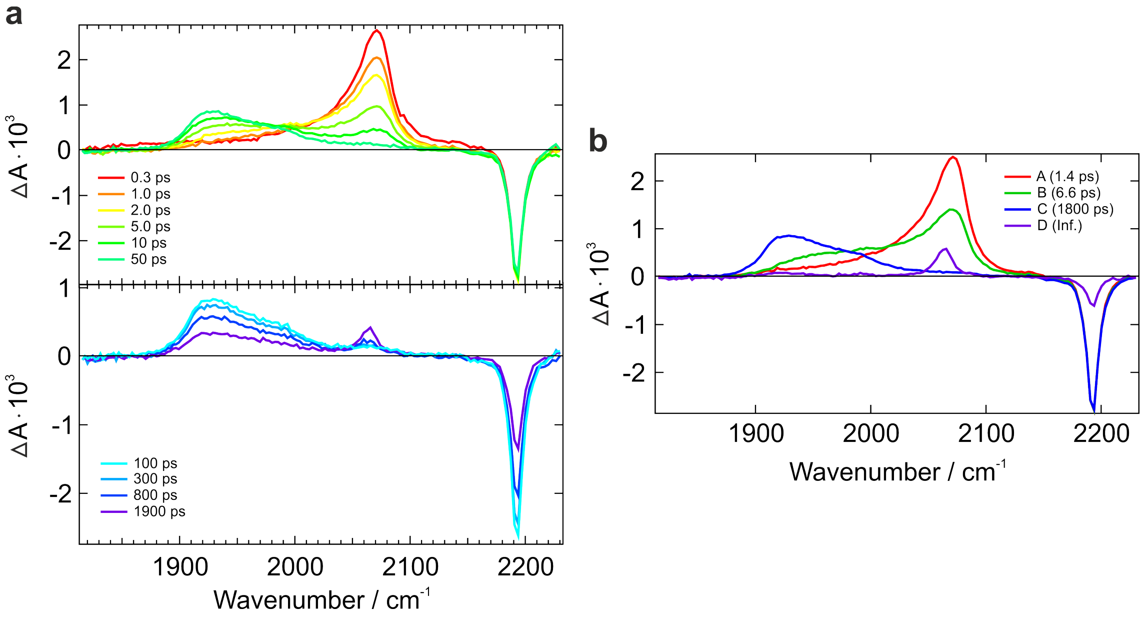

Supplementary Figure 9. a. TRIR spectral evolution upon 400 nm photoexcitation of **D** in the apolar cyclohexane. **b.** Evolution-associated difference spectra obtained from global analysis of TRIR data assuming A→B→C→D→ scheme.

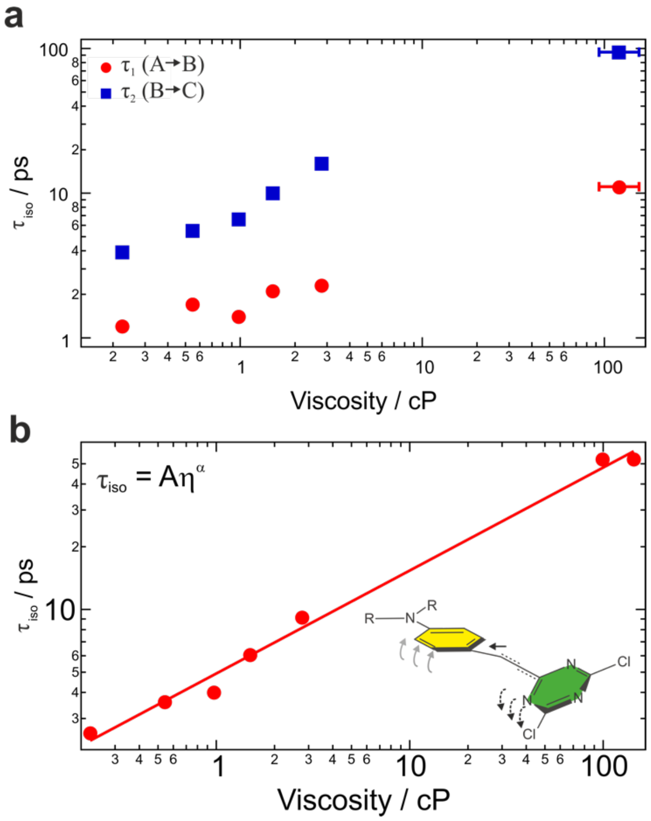

Supplementary Figure 10. a. Viscosity dependence of the time constants of the alkyne-allene isomerization of **D** in apolar aliphatic hydrocarbons obtained by using a sum of two exponential functions (τ_1 and τ_2 corresponding to A→B and B→C transformations in **Supplementary Figure 9b**). **b.** Alkyne-allene effective isomerization time constant as a function of viscosity along with the expected power-law fit.

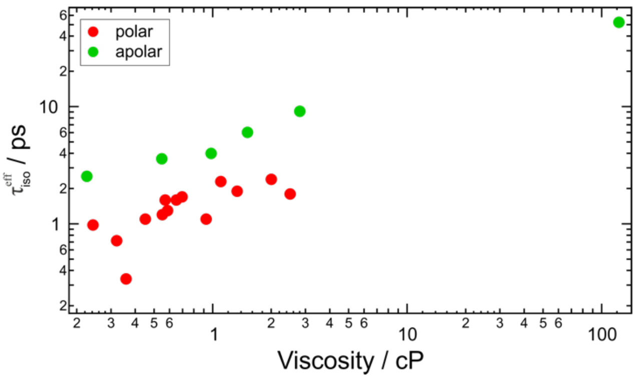

Supplementary Figure 11. Comparative viscosity trends for the effective isomerization time constant in apolar and polar solvent series.

Supplementary Table 8. Best-fit parameters recovered from the fit of Eq. (4) to the data presented in **Supplementary Figure 11**.

Solvent series	*A*	*α*
Apolar	5.0	0.49
Polar	1.6	0.41

It is important to characterize the final product of the reaction: the charge-separated allene species. Its geometry and electronic structure differ substantially depending on the dielectric properties of the environment. **Supplementary Figure 12** shows ground-state bleach-normalized transient IR spectra of the relaxed reaction product in several representative solvents of varying polarity: from the apolar cyclohexane to the highly polar propylene carbonate. Upon increasing reaction field of the environment, the spectral maximum upshifts substantially (from 1930 to 2020 cm^{-1}), narrows and loses its integral intensity. Such dramatic solvent dependence reflects change of the final product from a broad distribution of exciplex-like structures of intramolecularly coupled dialkylaniline radical cation and dichlorotriazine radical anion in apolar solvents to more decoupled localized ions on the opposite sides of the dyad in highly polar environments. In the first case, the electronic density in the π*-type orbital of the bridge sub-unit is higher than in the second resulting in downshifted frequency and stronger intensity.

This very different spectral appearance of the final product in various solvents reflects the varying nature of the species and not just the vibrational solvatochromism. **Supplementary Figure 13** shows that the vibrational solvatochromism of the alkyne bridging unit is noticeable (~10 cm^{-1} for S_0 (**Supplementary Figure 13a**) and up to ~20 cm^{-1} for S_1 (**Supplementary Figure 13b**)) but is not nearly close to the variation depicted in **Supplementary Figure 12**.

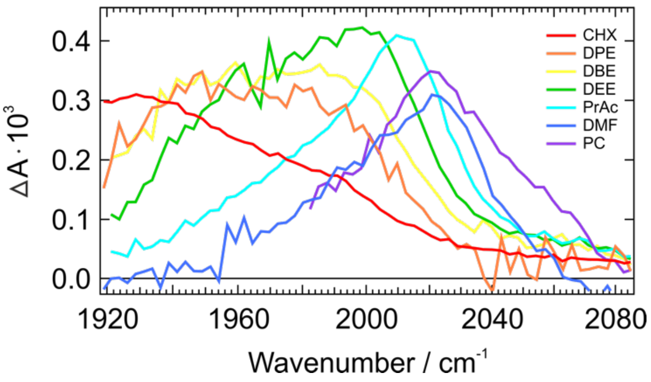

Supplementary Figure 12. GSB-normalized IR difference spectra of the final relaxed reaction product in several representative solvents of increasing polarity.

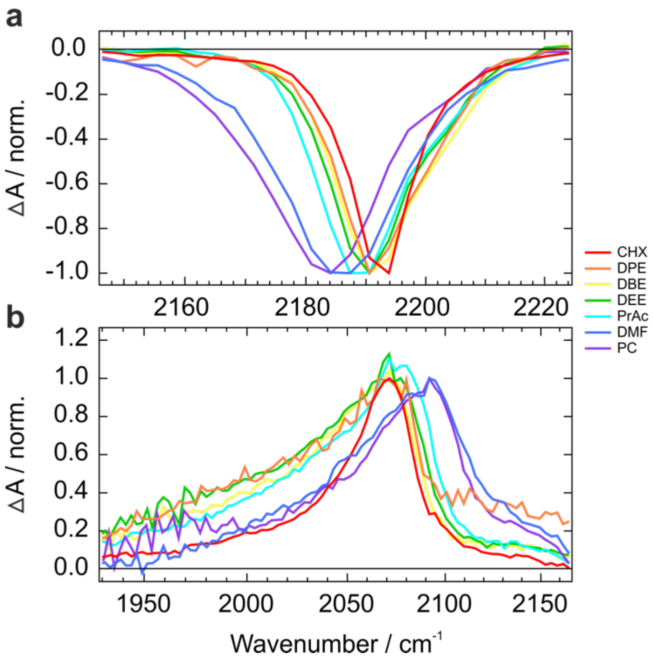

Supplementary Figure 13. a. Normalized IR difference spectra of the ground-state and **b.** Franck-Condon excited-state alkyne fragment in the same set of solvents as in **Supplementary Figure 12.**

Vibrational dynamics of O and Q

Supplementary Figure 14 shows evolution-associated spectra from a global analysis of the TRIR data of **O** in cyclohexane. Besides the small effect of vibrational cooling on the electronic band that was discussed in Section **Electronic excitonic transient absorption of O and Q** above, there is a noticeable appearance of a new vibrational band at ~1900 cm^{-1} happening on a ~50 ps timescale. To eliminate the broad and intense electronic resonance for reliable analysis of the vibrational dynamics in both apolar and polar media the following procedure was applied.

The ground-state bleach of the C≡C mode at ~2200 cm^{-1} for the earliest-time spectrum (usually at 200-300 fs) was fitted with a Lorentzian/Gaussian bandshape within the narrow frequency window (~100 cm^{-1} broad) and the underlying electronic absorption within the same window was fitted with a quadratic polynomial. The bleach contribution could then be

subtracted and the obtained bleach-free early-time spectrum was propagated in time by scaling with the time evolution of the excitonic band obtained as the average kinetics in the 2400-2500 cm^{-1} region that is free of vibrational resonances. The obtained matrix was subtracted from the rest of the spectrotemporal data matrix to yield double-difference pure vibrational TRIR spectra that represent the evolution of vibrational excited-state absorption features with respect to the initial early-time spectrum that itself represents the delocalized (octupolar or quadrupolar) excited state.

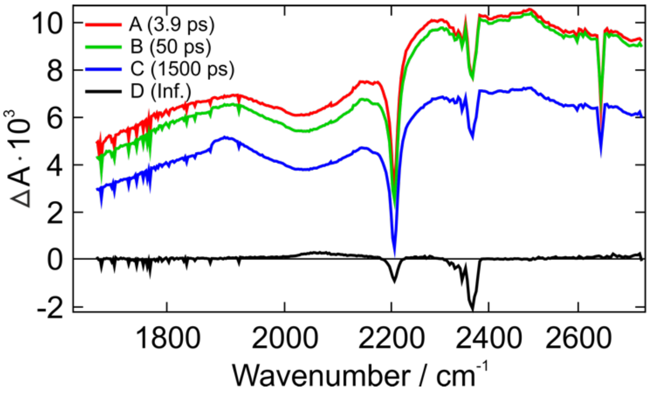

Supplementary Figure 14. Evolution-associated difference spectra of **O** obtained from global target analysis (A→B→C→D→) of the TRIR spectra in the apolar cyclohexane. EADS D represents the lowest triplet state featuring the alkyne C≡C absorption band at 2064 cm^{-1} and the absence of the excitonic band demonstrates that there is no coupling in the triplet manifold as expected according to the excitonic model.

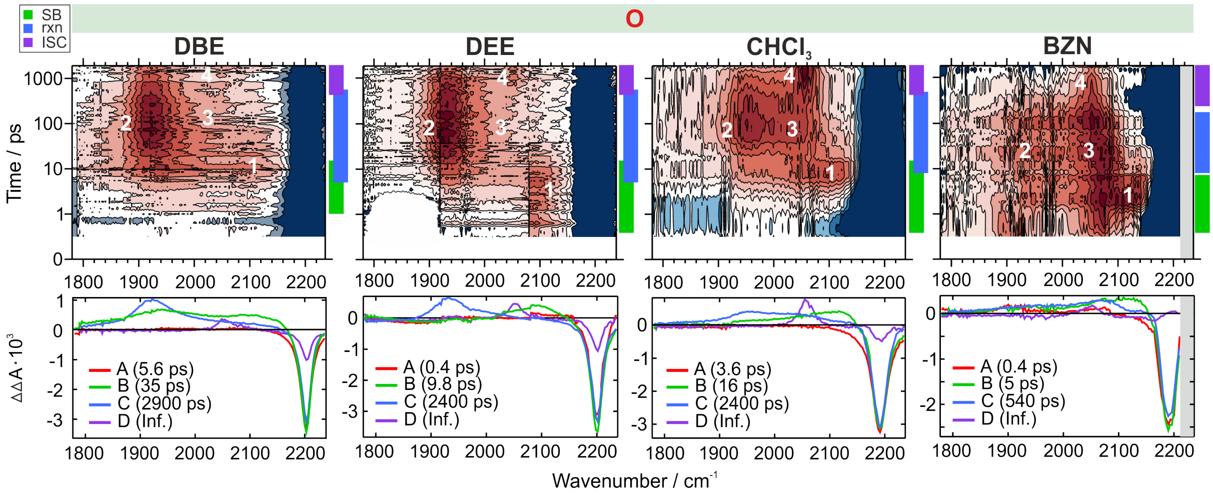

Supplementary Figure 15. Top. Contour maps of the double-difference TRIR spectra of **O** in several additional solvents of increasing polarity. Colorbars indicate dominant processes over specified timespan: SB (green), reaction (rxn, blue) or ISC (purple) **Bottom.** Evolution-associated double-difference spectra obtained from global target analysis (A→B→C→D→) of the data shown above.

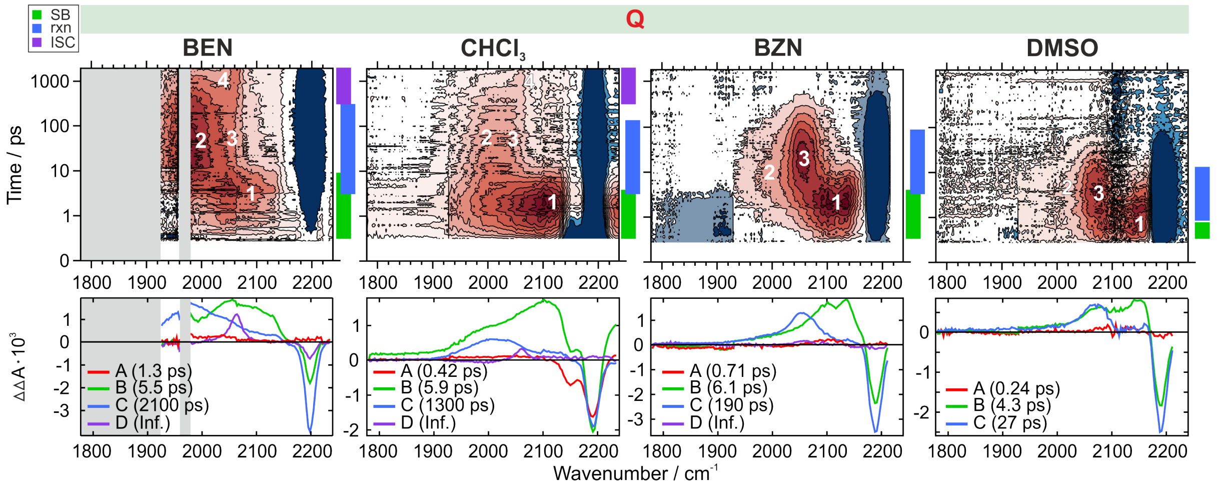

Supplementary Figure 16. Top. Contour maps of the double-difference TRIR spectra of **Q** in several additional solvents of increasing polarity. Colorbars indicate dominant processes over specified timespan: SB (green), reaction (rxn, blue) or ISC (purple). **Bottom.** Evolution-associated double-difference spectra obtained from global target analysis (A→B→C→D→) of the data shown above.

We carefully examined the dynamics of the GSB signal by means of bandshape analysis and observed some refill of the ground state population (~20% or less in all solvents, except for $CHCl_3$ where more noticeable refill takes place). However, this bleach refilling is not due to symmetry breaking and charge localization on a single arm. It is considerably lower than the expected 2/3 for the case of two branches being unaffected (and even discernibly lower than 1/3 for one unaffected branch). Additionally, similar fraction of the bleach refills in the quadrupolar system **Q**. Therefore, it rather trivially points out that internal conversion directs some of the molecules to the S_0 state lowering the quantum yield of the reaction from 100% observed for model **D**.

Supplementary Table 9. Ground-state bleach recovery parameters extracted from the IR lineshape analysis without taking into account triplet state population that results in the residual stationary amplitude of the bleach.

Solvent	O	Q
Cyclohexane	104 ps (8.4 %)<br>1.8 ns (91.6 %)	-
Benzene	8.9 ps (10 %)<br>3.2 ns (90 %)	2.1 ns
Di-n-butyl ether	61 ps (21.4 %)<br>2.9 ns (78.6 %)	-
Diethyl ether	16.5 ps (21.1 %)<br>3.54 ns (78.9 %)	-
Chloroform	23 ps (31.8 %)<br>2.4 ns (68.2 %)	2.1 ps (22 %)<br>38 ps (20 %)<br>1.3 ns (58 %)
THF	18.7 ps (22.2 %)	6.0 ps (13.7 %)

	1.6 ns (77.8 %)	622 ps (86.3 %)
Benzonitrile	21.2 ps (12.8 %)<br>540 ps (87.2 %)	205 ps
DMSO	-	0.39 ps (54 %)<br>28 ps (46 %)

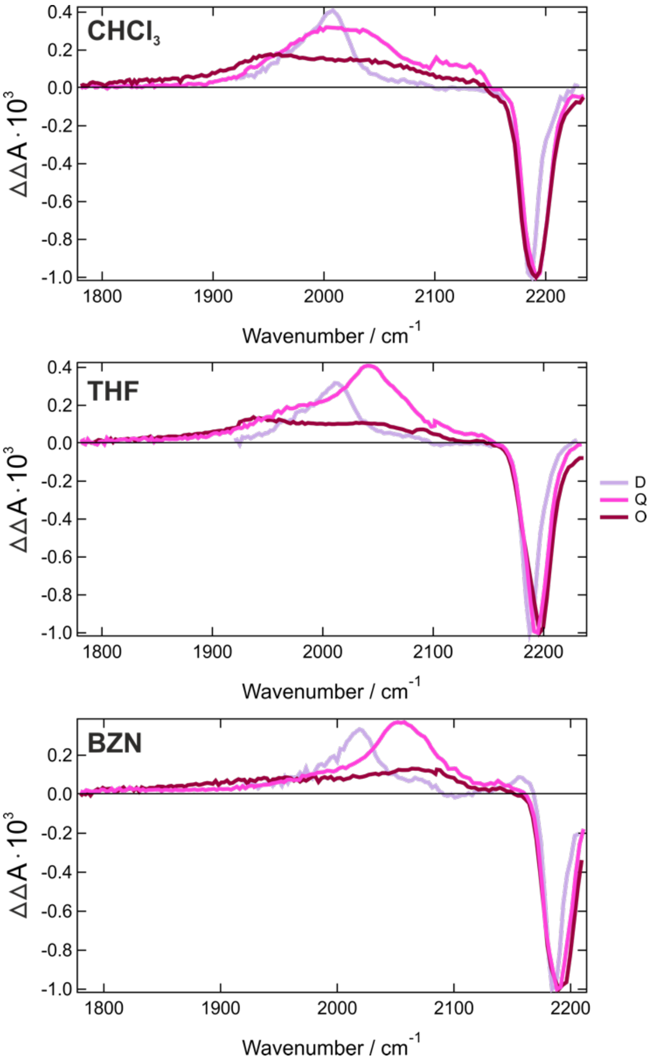

Supplementary Figure 17. Comparison of relaxed excited-state TRIR spectra of the dipolar, quadrupolar and octupolar systems in chloroform, THF and benzonitrile.

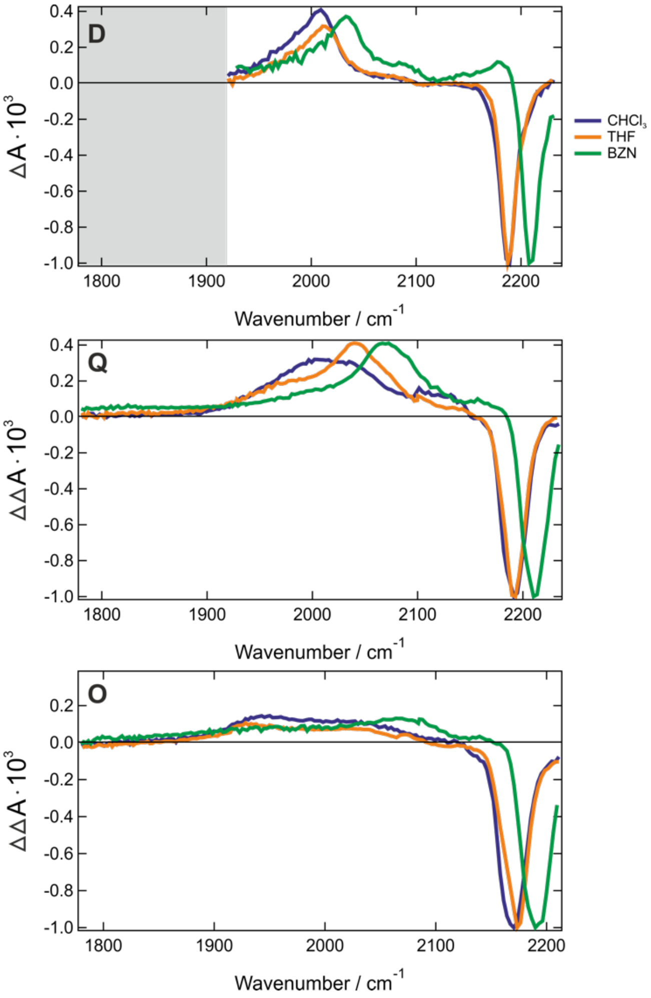

Supplementary Figure 18. Comparison of relaxed excited-state TRIR spectra of the dipolar, quadrupolar and octupolar systems in chloroform, THF and benzonitrile.

UV-vis and NIR TA

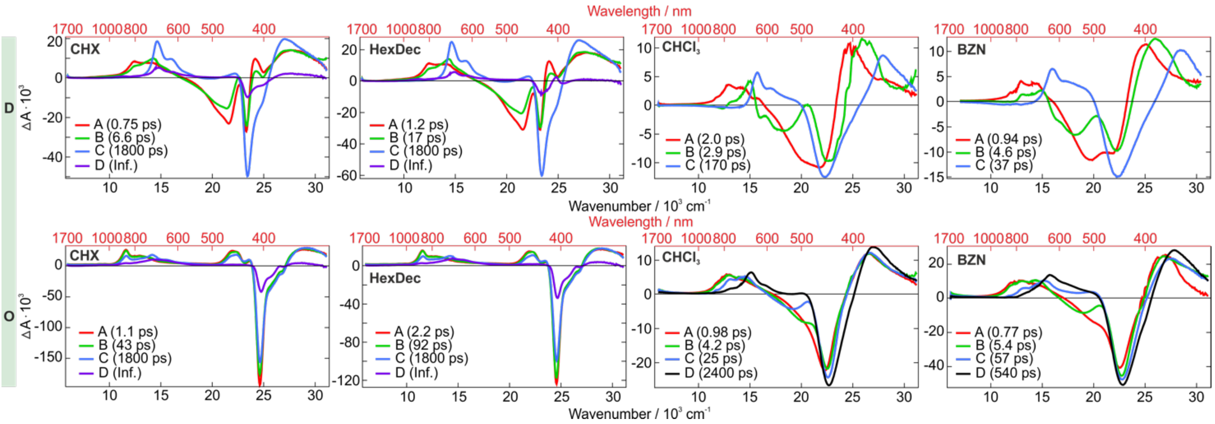

Supplementary Figure 19. Comparison of the evolution-associated difference spectra along with their timescales obtained from the analysis of the UV-vis-NIR TA data for **D** and **O** in various apolar and polar solvents.

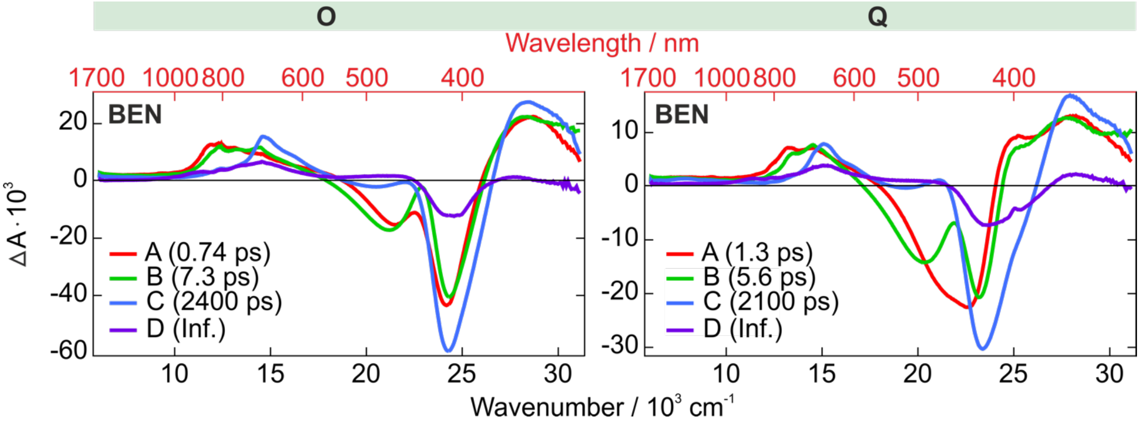

Supplementary Figure 20. Comparison of the evolution-associated difference spectra along with their timescales obtained from the analysis of the UV-vis-NIR TA data for **O** and **Q** in benzene.

Supplementary Figure 21 shows transient absorption spectra of the final fully relaxed excited state of the multipolar dyes compared to the **D** in the highly polar benzonitrile. The spectral signatures for all three molecules are very similar thus confirming that the charge separation reaction does take place in all three cases in an identical way. There is a notable difference though. The ground-state bleach signal between 400 and 500 nm becomes progressively more intense thus obscuring overlapping ESA and emission dynamics in the dipolar-quadrupolar-octupolar row of molecules (since the molar absorption coefficient scales with the number of branches).

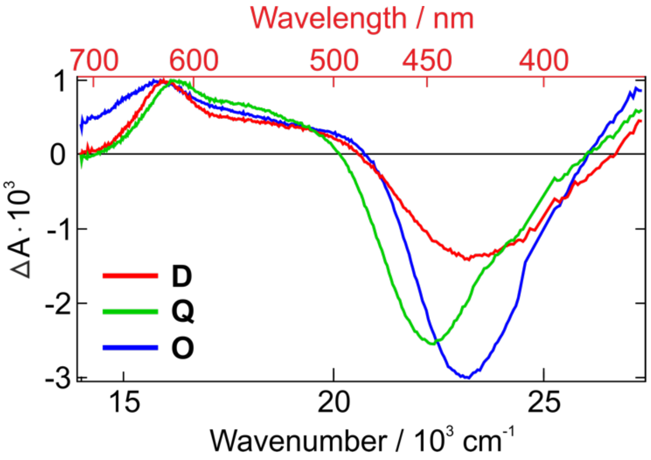

Supplementary Figure 21. Comparison of the TA signatures of the final relaxed species for the dipolar and multipolar dyes. The data are normalized to the maximum of the triazine radical anion absorption.

Therefore, we subtracted ground-state bleach contribution to the TA spectra (**Supplementary Figures 22-23, top**). For accurate subtraction of the full contribution of the featureless bleach signal the second-derivative method was used.[41] The resulting spectra contain only excited-state signatures: ESA and stimulated emission. Evolution-associated difference spectra obtained from the global analysis of these data are shown in

Supplementary Figures 22-23 (bottom). Again, we observe an overall similarity of the data for all three systems. The most important difference is the increase of the lifetime of the reaction product. There is a clear increase of the lifetime from the dipolar to the quadrupolar to the octupolar compound. **Supplementary Table 10** summarizes the time constants obtained from the global fitting for all the systems.

The other peculiar feature of the multipolar systems is the dominance of the excited-state absorption band at <460 nm. Even though the excited-state reaction leads to the reduction of the intensity of this band, it still dominates spectra even at later times. This high-energy feature can be associated with multitude of local overlapping transitions and thus is less useful than the low energy part of the transient spectrum.

Supplementary Table 10. UV-vis TA timescales extracted from the global target analysis of the bleach-refilled TA spectra (multipolar systems) or full TA spectra (dipolar system). First two timescales, τ_1 and τ_2 represent non-exponential solvation processes, whereas τ_3 corresponds to the allene excited-state lifetime of the molecule.

τ_i / ps	D	Q	O
		Chloroform	
τ_1	1.3	1.7	1.8
τ_2	2.0	4.4	5.8
τ_3	240	1700	2400
		THF	
τ_1	0.56	0.76	0.87
τ_2	0.73	3.3	4.8
τ_3	55	560	1600
		Benzonitrile	
τ_1	0.65	1.3	1.3
τ_2	3.1	5.7	5.6
τ_3	34	200	540
		DMSO	
τ_1	0.5	0.75	0.39
τ_2	1.8	3.1	2.9
τ_3	17	28	45

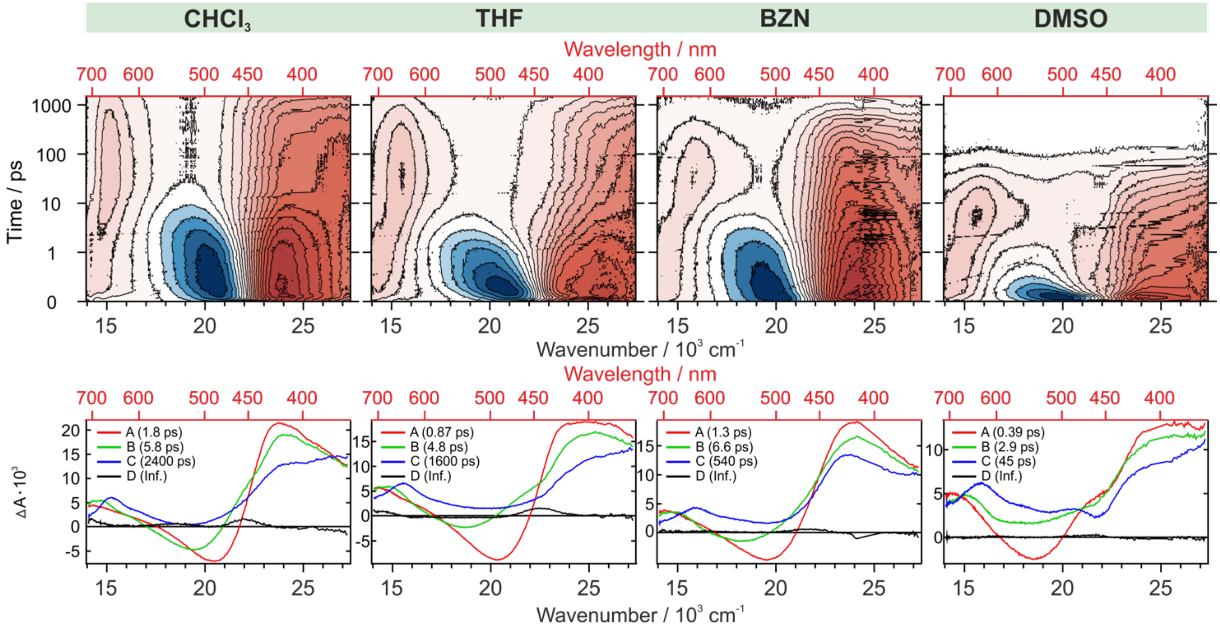

Supplementary Figure 22. Top. Excited-state TA spectra of **O** in solvents of growing polarity obtained after subtraction of the ground-state bleach contribution. The time axis is linear between 0 and 1 ps and logarithmic afterwards. Red color-coding is positive and corresponds to the excited-state absorption, blue is negative and corresponds to the stimulated emission signal. **Bottom.** Evolution-associated difference spectra obtained from the global target analysis (A→B→C→D→GS) of the data above.

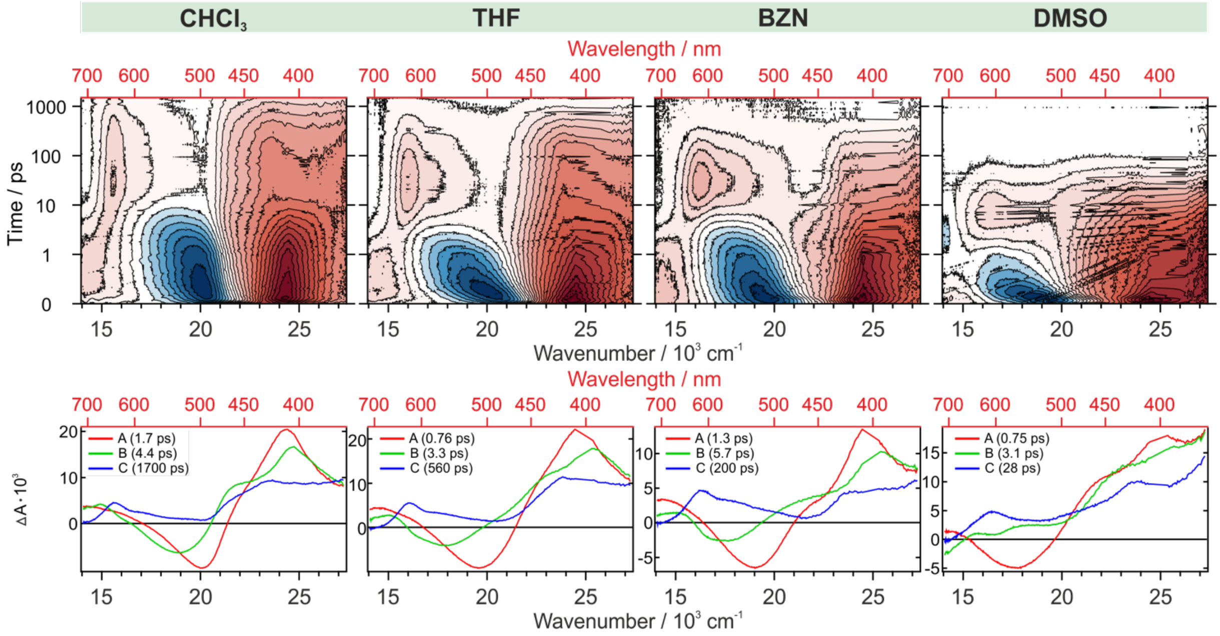

Supplementary Figure 23. Top. Excited-state TA spectra of **Q** in solvents of growing polarity obtained after subtraction of the ground-state bleach contribution. The time axis is linear between 0 and 1 ps and logarithmic afterwards. Red color-coding is positive and corresponds to the excited-state absorption, blue is negative and corresponds to the stimulated emission signal. **Bottom.** Evolution-associated difference spectra obtained from the global target analysis (A→B→C→GS) of the data above.

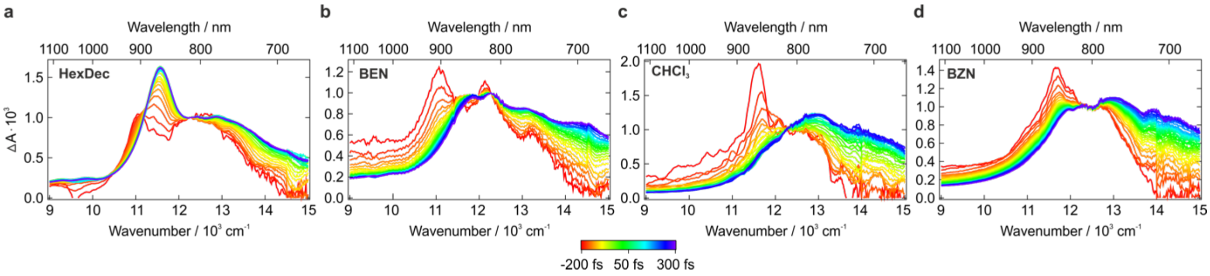

Supplementary Figure 24. Zooming into the very early-time (within the IRF) TA spectra of **O** in the NIR spectral region in apolar hexadecane (**a**) compared to polar solvents of increasing polarity (**b-d**). Spectra are normalized to the intensity at 12270 cm^{-1} (815 nm) to visualize the changing spectral shape. Extremely ultrafast decay of the sharp ESA marker band of higher excitonic transitions is evident in all polar solvents (**b-d**). The decay takes place within tens to few hundred fs (within the IRF, which is broader in this region compared to higher-energy UV-visible part of the spectrum due to the group velocity mismatch between 400 nm pump and NIR probe). Therefore, it is very challenging to detect this fleeting higher excitonic band except for apolar media (panel **a**) where it grows within the IRF and persists for the entire excited-state lifetime as discussed in the main text.

FLUPS

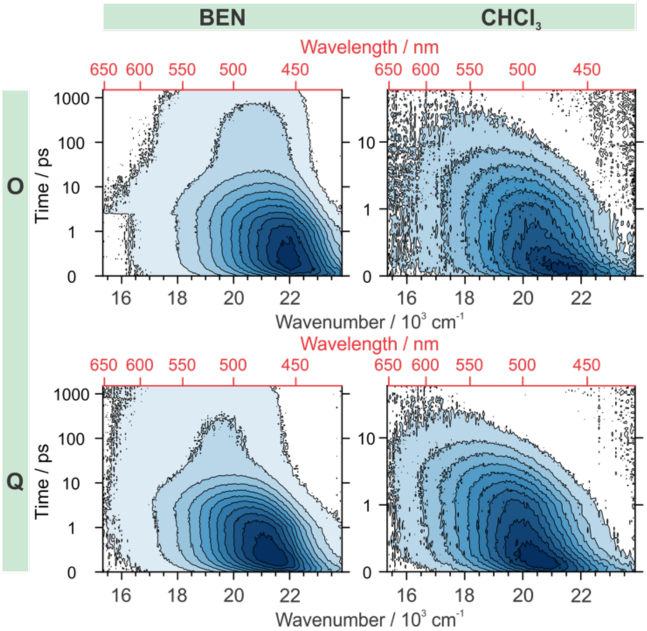

Supplementary Figure 25. FLUPS data contour maps for **O** and **Q** in benzene and chloroform. The time axis is linear between 0 and 1 ps and logarithmic afterwards.

The other electronic transition that is affected by SB is the downward $S_1 \rightarrow S_0$ one whose transition dipole decreases as was reported previously by our group.[42] FLUPS data were analyzed using two approaches: *i)* global target analyses similarly to other transient data (**Supplementary Figure 26**); ii) bandshape analysis of spectra at each time step (**Supplementary Table 11**). Since both SB and ensuing chemical reaction lead to fluorescence

quenching and the timescales of the processes are close and sometimes overlapping, it might be difficult to separate the processes cleanly. Nevertheless, EADS A represent the evolving delocalized exciton, EADS B is required to represent the nonexponential nature of solvation dynamics and does not correspond to a specific state but rather reflects the evolving symmetry broken exciton in the process of collapse onto a single arm, EADS C is the localized exciton, whereas EADS D represent the weakly to (almost) non-emissive photoproduct. Depending on the timescale of the isomerization/charge separation process the spectral signature of EADS C/D might represent a mixture of charge localization process and chemical reaction.

In general, the excitonic $S_2 \leftarrow S_1$ absorption is a more robust feature than $S_1 \rightarrow S_0$ fluorescence because it is applicable to non-fluorescing species as well and should generally appear in the less congested near- to mid-IR spectral range in contrast to the UV-visible range where fluorescence typically shows up. Also, it should suffer less from time-dependent shifting since time-dependent redshift of the fluorescence can originate from multiple various sources and is not specific to symmetry breaking phenomenon, whereas the shift of the excitonic $S_2 \leftarrow S_1$ absorption necessarily reflects on the change of interbranch molecular coupling.

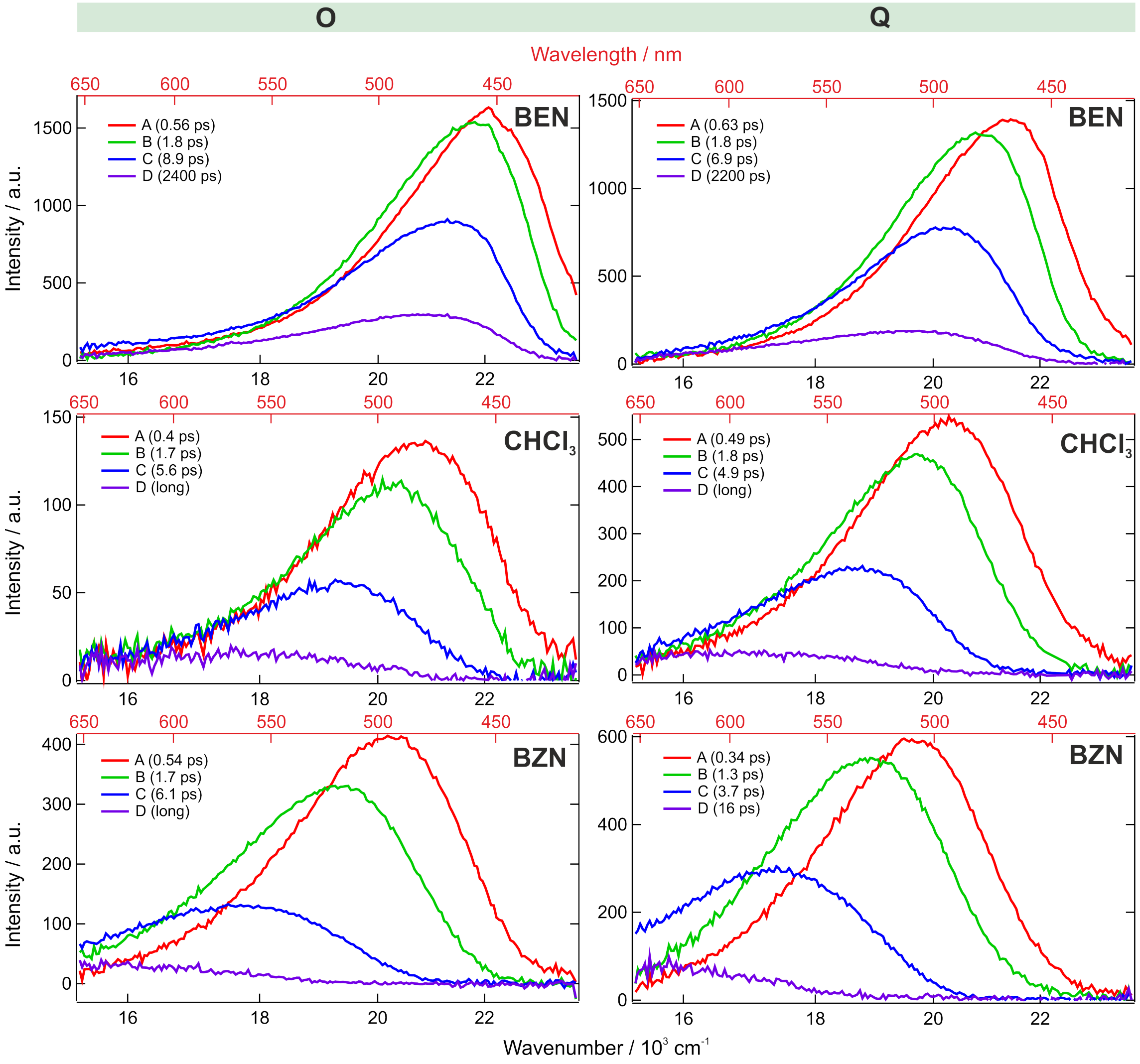

Supplementary Figure 26. Evolution-associated difference spectra obtained from the global target analysis of the FLUPS data for **O** and **Q** using A→B→C→D→GS scheme.

Supplementary Table 11. Results of the bandshape analysis of the FLUPS data for the multipolar dyes. Lognorm was used as a lineshape function.

Parameter	O			Q		
	Benzene	Chloroform	Benzonitrile	Benzene	Chloroform	Benzonitrile
**Area**	3.1 ps (44 %)<br>11 ps (35 %)<br>2.44 ns (21 %)	0.63 ps (31 %)<br>3.7 ps (55 %)<br>70 ps (14 %)	1.9 ps (64 %)<br>9.0 ps (30 %)<br>Inf. (6 %)	3.6 ps (60 %)<br>11 ps (24 %)<br>2.34 ns (15 %)	0.84 ps (25 %)<br>3.9 ps (67 %)<br>6.9 ps (9 %)	2.2 ps (40 %)<br>4.5 ps (46 %)<br>21 ps (14 %)
**Peakshift**	**22120-20800 = 1320 cm^{-1}**<br>0.8 ps (47 %)<br>10 ps (44 %)<br>170 ps (9 %)	**20930-17300 = 3230 cm^{-1}**<br>0.35 ps (19 %)<br>3.5 ps (27 %)<br>23 ps (54 %)	**20360-15930 = 4430 cm^{-1}**<br>0.23 ps (31 %)<br>6.1 ps (69 %)	**21350-19610 = 1740 cm^{-1}**<br>0.16 ps (29 %)<br>1.3 ps (31 %)<br>12.3 ps (40 %)	**20460-17270 = 3190 cm^{-1}**<br><0.10 ps (4 %)<br>0.39 ps (25 %)<br>8.5 ps (71 %)	**19970-15890 = 4080 cm^{-1}**<br>0.23 ps (32 %)<br>4.2 ps (68 %)

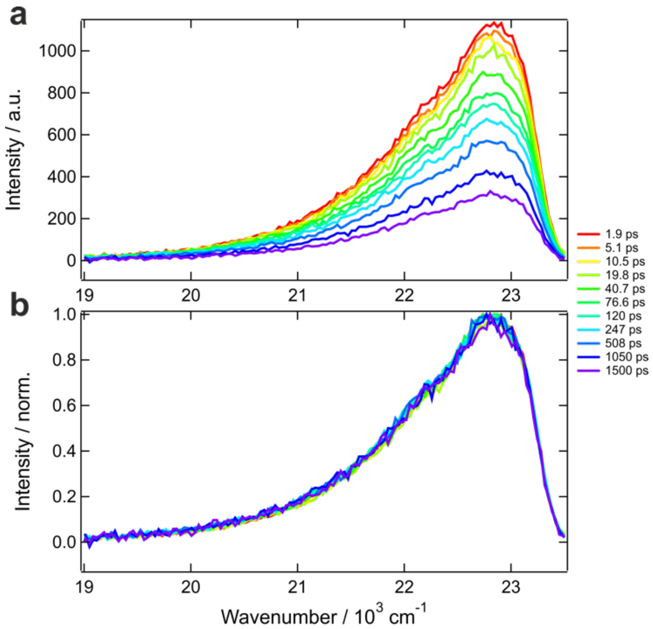

Supplementary Figure 27. Spectral evolution of the time-resolved fluorescence spectra of **O** in apolar cyclohexane (**a**) and intensity-normalized spectra (**b**). Only the lower-energy part of the spectrum is shown since the higher-energy part was inaccessible due to the spectral limitation of the FLUPS instrument under current excitation conditions. The dip on the blue edge of the spectra is due to the 420 nm spectral filter used to cut out the scattering of the excitation source.

SUPPLEMENTARY REFERENCES

(1) Dereka, B.; Svechkarev, D.; Rosspeintner, A.; Tromayer, M.; Liska, R.; Mohs, A. M.; Vauthey, E. Direct Observation of a Photochemical Alkyne–Allene Reaction and of a Twisted and Rehybridized Intramolecular Charge-Transfer State in a Donor–Acceptor Dyad. *J. Am. Chem. Soc.* **2017**, *139* (46), 16885–16893.

(2) Zhang, L.; Zou, L.; Xiao, J.; Zhou, P.; Zhong, C.; Chen, X.; Qin, J.; Mariz, I. F. A.; Maçôas, E. Symmetrical and Unsymmetrical Multibranched D-Pi-A Molecules Based on 1,3,5-Triazine Unit: Synthesis and Photophysical Properties. *J. Mater. Chem.* **2012**, *22* (33), 16781.

(3) Li, Z.; Siklos, M.; Pucher, N.; Cicha, K.; Ajami, A.; Husinsky, W.; Rosspeintner, A.; Vauthey, E.; Gescheidt, G.; Stampfl, J.; Liska, R. Synthesis and Structure-Activity Relationship of Several Aromatic Ketone-Based Two-Photon Initiators. *J. Polym. Sci. Part A Polym. Chem.* **2011**, *49* (17), 3688–3699.

(4) Pucher, N.; Rosspeintner, A.; Satzinger, V.; Schmidt, V.; Gescheidt, G.; Stampfl, J.; Liska, R. Structure–Activity Relationship in D-π-A-π-D-Based Photoinitiators for the Two-Photon-Induced Photopolymerization Process. *Macromolecules* **2009**, *42* (17), 6519–6528.

(5) Sonoda, M.; Inaba, A.; Itahashi, K.; Tobe, Y. Synthesis of Differentially Substituted Hexaethynylbenzenes Based on Tandem Sonogashira and Negishi Cross-Coupling Reactions. *Org. Lett.* **2001**, *3* (15), 2419–2421.

(6) Braml, N. E.; Stegbauer, L.; Lotsch, B. V.; Schnick, W. Synthesis of Triazine-Based Materials by Functionalization with Alkynes. *Chem. - A Eur. J.* **2015**, *21* (21), 7866–7873.

(7) Gardecki, J. A.; Maroncelli, M. Set of Secondary Emission Standards for Calibration of the Spectral Responsivity in Emission Spectroscopy. *Appl. Spectrosc.* **1998**, *52* (9), 1179–1189.

(8) Makarov, N. S.; Drobizhev, M.; Rebane, A. Two-Photon Absorption Standards in the 550-1600 Nm Excitation Wavelength Range. *Opt. Express* **2008**, *16* (6), 4029.

(9) Muller, P.-A.; Högemann, C.; Allonas, X.; Jacques, P.; Vauthey, E. Deuterium Isotope Effect on the Charge Recombination Dynamics of Contact Ion Pairs Formed by Electron-Transfer Quenching in Acetonitrile. *Chem. Phys. Lett.* **2000**, *326* (3–4), 321–327.

(10) Söderberg, M.; Dereka, B.; Marrocchi, A.; Carlotti, B.; Vauthey, E. Ground-State Structural Disorder and Excited-State Symmetry Breaking in a Quadrupolar Molecule. *J. Phys. Chem. Lett.* **2019**, *10* (11), 2944–2948.

(11) Bredenbeck, J.; Hamm, P. Versatile Small Volume Closed-Cycle Flow Cell System for Transient Spectroscopy at High Repetition Rates. *Rev. Sci. Instrum.* **2003**, *74* (6), 3188–3189.

(12) Lang, B. Photometrics of Ultrafast and Fast Broadband Electronic Transient Absorption Spectroscopy: State of the Art. *Rev. Sci. Instrum.* **2018**, *89* (9), 093112.

(13) Banerji, N.; Duvanel, G.; Perez-Velasco, A.; Maity, S.; Sakai, N.; Matile, S.; Vauthey, E. Excited-State Dynamics of Hybrid Multichromophoric Systems: Toward an

Excitation Wavelength Control of the Charge Separation Pathways. *J. Phys. Chem. A* **2009**, *113* (29), 8202–8212.

(14) Tokunaga, E.; Terasaki, A.; Kobayashi, T. Femtosecond Continuum Interferometer for Transient Phase and Transmission Spectroscopy. *J. Opt. Soc. Am. B* **1996**, *13* (3), 496.

(15) DeFlores, L. P.; Nicodemus, R. a; Tokmakoff, A. Two-Dimensional Fourier Transform Spectroscopy in the Pump-Probe Geometry. *Opt. Lett.* **2007**, *32* (20), 2966.

(16) Demirdöven, N.; Khalil, M.; Golonzka, O.; Tokmakoff, A. Dispersion Compensation with Optical Materials for Compression of Intense Sub-100-Fs Mid-Infrared Pulses. *Opt. Lett.* **2002**, *27* (6), 433.

(17) Zhang, X.-X.; Wurth, C.; Zhao, L.; Resch-Genger, U.; Ernsting, N. P.; Sajadi, M. Femtosecond Broadband Fluorescence Upconversion Spectroscopy: Improved Setup and Photometric Correction. *Rev. Sci. Instrum.* **2011**, *82* (6), 063108.

(18) Zhao, L.; Luis Pérez Lustres, J.; Farztdinov, V.; Ernsting, N. P. Femtosecond Fluorescence Spectroscopy by Upconversion with Tilted Gate Pulses. *Phys. Chem. Chem. Phys.* **2005**, *7* (8), 1716–1725.

(19) Gerecke, M.; Bierhance, G.; Gutmann, M.; Ernsting, N. P.; Rosspeintner, A. Femtosecond Broadband Fluorescence Upconversion Spectroscopy: Spectral Coverage versus Efficiency. *Rev. Sci. Instrum.* **2016**, *87* (5), 053115.

(20) Zhang, X.-X.; Würth, C.; Zhao, L.; Resch-Genger, U.; Ernsting, N. P.; Sajadi, M. Femtosecond Broadband Fluorescence Upconversion Spectroscopy: Improved Setup and Photometric Correction. *Rev. Sci. Instrum.* **2011**, *82* (6), 063108.

(21) Frisch, M. J.; Trucks, G. W.; Schlegel, H. B.; Scuseria, G. E.; Robb, M. A.; Cheeseman, J. R.; Scalmani, G.; Barone, V.; Mennucci, B.; Petersson, G. A.; Nakatsuji, H.; Caricato, M.; Li, X.; Hratchian, H. P.; Izmaylov, A. F.; Bloino, J.; Zheng, G.; Sonnenberg, J. L.; Hada, M.; Ehara, M.; Toyota, K.; Fukuda, R.; Hasegawa, J.; Ishida, M.; Nakajima, T.; Honda, Y.; Kitao, O.; Nakai, H.; Vreven, T.; Montgomery, Jr., J. A.; Peralta, J. E.; Ogliaro, F.; Bearpark, M.; Heyd, J. J.; Brothers, E.; Kudin, K. N.; Staroverov, V. N.; Kobayashi, R.; Normand, J.; Raghavachari, K.; Rendell, A.; Burant, J. C.; Iyengar, S. S.; Tomasi, J.; Cossi, M.; Rega, N.; Millam, J. M.; Klene, M.; Knox, J. E.; Cross, J. B.; Bakken, V.; Adamo, C.; Jaramillo, J.; Gomperts, R.; Stratmann, R. E.; Yazyev, O.; Austin, A. J.; Cammi, R.; Pomelli, C.; Ochterski, J. W.; Martin, R. L.; Morokuma, K.; Zakrzewski, V. G.; Voth, G. A.; Salvador, P.; Dannenberg, J. J.; Dapprich, S.; Daniels, A. D.; Farkas, Ö.; Foresman, J. B.; Ortiz, J. V.; Cioslowski, J.; Fox, D. J. Gaussian 09 (Revision D.1). Gaussian, Inc.: Wallingford CT 2009.

(22) van Stokkum, I. H. M.; Larsen, D. S.; van Grondelle, R. Global and Target Analysis of Time-Resolved Spectra. *Biochim. Biophys. Acta - Bioenerg.* **2004**, *1657* (2–3), 82–104.

(23) Ruckebusch, C.; Sliwa, M.; Pernot, P.; de Juan, A.; Tauler, R. Comprehensive Data Analysis of Femtosecond Transient Absorption Spectra: A Review. *J. Photochem. Photobiol. C Photochem. Rev.* **2012**, *13* (1), 1–27.

(24) Tromayer, M.; Gruber, P.; Rosspeintner, A.; Ajami, A.; Husinsky, W.; Plasser, F.; González, L.; Vauthey, E.; Ovsianikov, A.; Liska, R. Wavelength-Optimized Two-Photon Polymerization Using Initiators Based on Multipolar Aminostyryl-1,3,5-Triazines. *Sci. Rep.* **2018**, *8* (1), 17273.

(25) Kamlet, M. J.; Abboud, J. L.; Taft, R. W. The Solvatochromic Comparison Method. 6. The Π* Scale of Solvent Polarities1. *J. Am. Chem. Soc.* **1977**, *99* (18), 6027–6038.

(26) Dereka, B.; Rosspeintner, A.; Krzeszewski, M.; Gryko, D. T.; Vauthey, E. Symmetry-Breaking Charge Transfer and Hydrogen Bonding: Toward Asymmetrical Photochemistry. *Angew. Chemie Int. Ed.* **2016**, *55* (50), 15624–15628.

(27) Dereka, B.; Vauthey, E. Solute–Solvent Interactions and Excited-State Symmetry Breaking: Beyond the Dipole–Dipole and the Hydrogen-Bond Interactions. *J. Phys. Chem. Lett.* **2017**, *8* (16), 3927–3932.

(28) Dereka, B.; Rosspeintner, A.; Stężycki, R.; Ruckebusch, C.; Gryko, D. T.; Vauthey, E. Excited-State Symmetry Breaking in a Quadrupolar Molecule Visualized in Time and Space. *J. Phys. Chem. Lett.* **2017**, *8*, 6029–6034.

(29) Suppan, P.; Ghoneim, N. *Solvatochromism*; Royal Society of Chemistry: Cambridge, 1997.

(30) McRae, E. G. Theory of Solvent Effects on Molecular Electronic Spectra. Frequency Shifts. *J. Phys. Chem.* **1957**, *61* (5), 562–572.

(31) Fried, S. D.; Boxer, S. G. Measuring Electric Fields and Noncovalent Interactions Using the Vibrational Stark Effect. *Acc. Chem. Res.* **2015**, *48* (4), 998–1006.

(32) Schneider, S. H.; Kratochvil, H. T.; Zanni, M. T.; Boxer, S. G. Solvent-Independent Anharmonicity for Carbonyl Oscillators. *J. Phys. Chem. B* **2017**, *121* (10), 2331–2338.

(33) Abrarov, S. M.; Quine, B. M. Efficient Algorithmic Implementation of the Voigt/Complex Error Function Based on Exponential Series Approximation. *Appl. Math. Comput.* **2011**, *218* (5), 1894–1902.

(34) Thielges, M. C.; Fayer, M. D. Time-Dependent Fifth-Order Bands in Nominally Third-Order 2D IR Vibrational Echo Spectra. *J. Phys. Chem. A* **2011**, *115* (34), 9714–9723.

(35) Kraack, J. P.; Hamm, P. Vibrational Ladder-Climbing in Surface-Enhanced, Ultrafast Infrared Spectroscopy. *Phys. Chem. Chem. Phys.* **2016**, *18* (24), 16088–16093.

(36) Ojeda, J.; Arrell, C. A.; Longetti, L.; Chergui, M.; Helbing, J. Charge-Transfer and Impulsive Electronic-to-Vibrational Energy Conversion in Ferricyanide: Ultrafast Photoelectron and Transient Infrared Studies. *Phys. Chem. Chem. Phys.* **2017**, *19* (26), 17052–17062.

(37) Hamm, P.; Zanni, M. T. *Concepts and Methods of 2D Infrared Spectroscopy*; Cambridge University Press: New York, 2011.

(38) Horng, M. L.; Gardecki, J. a.; Papazyan, A.; Maroncelli, M. Subpicosecond Measurements of Polar Solvation Dynamics: Coumarin 153 Revisited. *J. Phys. Chem.* **1995**, *99* (48), 17311–17337.

(39) Velsko, S. P.; Fleming, G. R. Solvent Influence on Photochemical Isomerizations: Photophysics of DODCI. *Chem. Phys.* **1982**, *65* (1), 59–70.

(40) Sundström, V.; Gillbro, T.; Bergström, H. Picosecond Kinetics of Radiationless Relaxations of Triphenyl Methane Dyes. Evidence for a Rapid Excited-State Equilibrium between States of Differing Geometry. *Chem. Phys.* **1982**, *73* (3), 439–458.

(41) Wilcken, R.; Schildhauer, M.; Rott, F.; Huber, L. A.; Guentner, M.; Thumser, S.; Hoffmann, K.; Oesterling, S.; de Vivie-Riedle, R.; Riedle, E.; Dube, H. Complete Mechanism of Hemithioindigo Motor Rotation. *J. Am. Chem. Soc.* **2018**, *140* (15), 5311–5318.

(42) Beckwith, J. S.; Rosspeintner, A.; Licari, G.; Lunzer, M.; Holzer, B.; Fröhlich, J.; Vauthey, E. Specific Monitoring of Excited-State Symmetry Breaking by Femtosecond Broadband Fluorescence Upconversion Spectroscopy. *J. Phys. Chem. Lett.* **2017**, 5878–5883.
